# Key intermediates and Cu active sites for CO_2_ electroreduction to ethylene and ethanol

**DOI:** 10.1038/s41560-024-01633-4

**Published:** 2024-09-11

**Authors:** Chao Zhan, Federico Dattila, Clara Rettenmaier, Antonia Herzog, Matias Herran, Timon Wagner, Fabian Scholten, Arno Bergmann, Núria López, Beatriz Roldan Cuenya

**Affiliations:** 1 Department of Interface Science, Fritz-Haber Institute of the Max-Planck Society, Berlin, Germany; 2 Institute of Chemical Research of Catalonia (ICIQ-CERCA), The Barcelona Institute of Science and Technology (BIST), Tarragona, Spain; 3 Department of Applied Science and Technology (DISAT), Politecnico di Torino, Turin, Italy

**Keywords:** Electrocatalysis, Energy science and technology

## Abstract

Electrochemical reduction of CO_2_ (CO_2_RR) to multi-carbon products is a promising technology to store intermittent renewable electricity into high-added-value chemicals and close the carbon cycle. Its industrial scalability requires electrocatalysts to be highly selective to certain products, such as ethylene or ethanol. However, a substantial knowledge gap prevents the design of tailor-made materials, as the properties ruling the catalyst selectivity remain elusive. Here we combined in situ surface-enhanced Raman spectroscopy and density functional theory on Cu electrocatalysts to unveil the reaction scheme for CO_2_RR to C_2+_ products. Ethylene generation occurs when *OC–CO(H) dimers form via CO coupling on undercoordinated Cu sites. The ethanol route opens up only in the presence of highly compressed and distorted Cu domains with deep *s*-band states via the crucial intermediate *OCHCH_2_. By identifying and tracking the critical intermediates and specific active sites, our work provides guidelines to selectively decouple ethylene and ethanol production on rationally designed catalysts.

## Abstract (web summary)

Electrochemical reduction of CO_2_ can yield many different products; a better understanding of the key mechanisms at play is needed to guide the design of selective catalysts. Here the authors use in situ surface-enhanced Raman spectroscopy and simulations to elucidate reaction schemes for CO_2_ reduction to ethylene and ethanol.

## Main

Electrochemical reduction of CO_2_ (CO_2_RR) to chemicals is a promising approach to store intermittent renewable energy as valuable fuels and feedstocks^1,2^. In particular, the synthesis of multi-carbon (C_2+_) products, such as ethylene and ethanol, is highly attractive because of their versatility in the chemical and energy industries^3^, and processes involving C–C bond formation are of great relevance from the point of view of fundamental research^2,4^.

Copper-based materials are the most selective catalysts for electrochemically reducing CO_2_ to C_2+_ products^2^. A myriad of studies have focused on understanding the C–C coupling mechanism, and although some consensus has been reached, the dependence of these paths on the particular material history is yet to be confirmed^5,6^. Several coupling pathways have been proposed^7,8^, suggesting adsorbed CO (*CO) as the building block of multi-carbon products^5,9,10^ via dimerization (OC–CO)^7^ or coupling (OC–CHO or OC–COH)^8^ steps. Experimentally, *CO has been detected on Cu surfaces using in situ Raman and infrared spectroscopy during CO_2_RR^6,11,12^, and a direct correlation between CO coverage and selectivity towards C_2+_ products exists^13^. Recently, possible C–C coupling intermediates *OCCO or *OCCOH were detected by infrared spectroscopy during the electrochemical reduction of CO (COR)^14,15^. However, while CO coupling has been repeatedly confirmed, observing and tracking potential-dependent CO dimerization and intermediates during the subsequent gradual reduction process towards final products has not yet been achieved.

Among the CO_2_RR reaction intermediates^7,8^, *OCHCH_2_ is considered the last common precursor in the ethylene and ethanol routes, although experimental confirmation is still missing^2,7^. In fact, glyoxal and glycolaldehyde reduce to acetaldehyde, ethanol and ethylene glycol only on polycrystalline copper^16–18^, suggesting an exclusive ethanol-selective route via *OCHCH_2_. Recently, a methyl carbonyl intermediate, *OCCH_3_, has been proposed as an alternative intermediate for ethanol and propanol^19,20^. In general, the reaction pathways for ethylene and ethanol formation are defined by logical intuition based on experimental and computational results^7,8,16,21^. However, the lack of robust experimental evidence hinders the rational design of CO_2_RR electrocatalysts, limiting the overall energy efficiency of the process.

The dynamics of electrocatalysts following charge accumulation, redox chemistry and the presence of adsorbates which can structurally deteriorate the catalyst surface lead to a very complex behaviour^4^. Thus, the generalization of insights gathered through model interfaces in an ultrahigh vacuum (UHV) system needs to be compared with the in situ electrocatalytic solid–liquid interface^22^. So far, a direct link between the local atomic surface structure and the Cu coordination environment on the catalytic process and, in particular, its influence on distinct reaction pathways remains unclear. CO strongly deteriorates the Cu surface and leads to undercoordinated sites^23^, which has a significant influence on CO_2_RR. For example, by comparing various single-crystal surfaces with different surface roughness, we previously showed that undercoordinated Cu sites are required for C_2_ formation^24^. However, it is important to further close this knowledge gap by unravelling how Cu coordination and local stress under reaction conditions affect the catalytic behaviour and thus the full mechanistic picture. To this end, it is required to overcome the challenging low surface coverages of intermediates due to their short lifetimes as well as disentangle the similarities in the vibrational properties of different C–C coupling intermediates^14^.

Here we provide direct spectroscopic evidence of potential-dependent CO dimerization and subsequent reduction intermediates during CO_2_RR on a Cu surface by combining insights from in situ surface-enhanced Raman spectroscopy (SERS) and density functional theory (DFT)-based vibrational analysis. This allows us to identify crucial Cu sites and surface intermediates for either ethylene or ethanol, leading to the definition of an updated reaction scheme for CO_2_RR to C_2_ products.

## Electrode preparation and CO_2_RR test

**Fig. 1:**
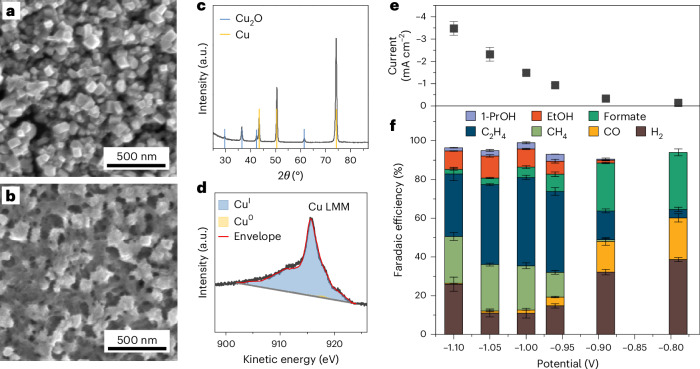
Characterization of the morphology of electrochemically treated Cu foil electrode and its CO_2_RR performance. **a**,**b**, SEM images of an as-prepared (electrochemically treated) Cu foil electrode acquired before (**a**) and after (**b**) CO_2_RR in CO_2_-saturated 0.1 M KHCO_3_ electrolyte at −1.0 V_RHE_ for 1 h. **c**, XRD pattern of the as-prepared Cu foil electrode. **d**, Cu LMM XAES spectrum of the as-prepared Cu foil electrode. **e**, Potential-dependent current density. **f**, Faradaic efficiency of the electrochemically treated Cu foil electrode in CO_2_-saturated 0.1 M KHCO_3_ electrolyte for 1 h. The error bars in **e** and **f** correspond to the s.d. of three independent measurements. Data are given as average ± s.d. Source data

The Cu electrodes were prepared by an electrochemical oxidation–reduction process (see Methods for details). Scanning electron microscopy (SEM) images show the formation of cubic structures with high density (Fig. 1a,b and Supplementary Fig. 1). The X-ray diffraction (XRD) pattern confirms the presence of Cu_2_O (Fig. 1c). Cu LMM X-ray Auger electron spectroscopy (XAES) data indicate a surface composition of about 94% Cu^I^ and 6% Cu^0^ species (Fig. 1d and Supplementary Fig. 2), while further elemental analysis confirmed the absence of other elements, such as Na, Cl and S (Supplementary Fig. 3). After 1 h of CO_2_RR at −1.0 V_RHE_ (where RHE is reversible hydrogen electrode) in 0.1 M KHCO_3_, the XRD pattern of Cu_2_O almost completely disappeared (Supplementary Fig. 4), and the cubic morphology partially changed (Fig. 1b), consistent with previous reports^13^. In spite of these morphological changes, no unexpected elements (for example, K) were present in post-catalysis X-ray photoelectron spectroscopy (XPS) analysis (Supplementary Fig. 5). Thus, we opted to use this treatment to obtain sufficient enhancement of the Raman signals.

Similar to the oxide-derived Cu (OD-Cu) materials, which are selective for C–C coupling^24–27^, the as-prepared Cu electrode with a high electrochemical surface roughness factor (14.6; Supplementary Fig. 6, electropolished Cu foil as a reference) shows high performance towards C_2+_ products and provides the opportunity to detect C–C coupling intermediates^25^. The specific current densities increase up to at least −1.1 V (Fig. 1e), while the Faradaic efficiencies of CO_2_RR products and H_2_ vary strongly with the applied potential (Fig. 1f). The Faradaic efficiencies of C_2+_ products show a typical volcano dependence on the applied potential, with a maximum of 65% at about −1.0 V_RHE_. The onset potentials for ethylene and ethanol are about −0.8 V_RHE_ and −0.9 V_RHE_, with these products reaching a maximum value at −1.0 V_RHE_ and −1.05 V_RHE_, respectively.

## Potential-dependent intermediate evolution

**Fig. 2:**
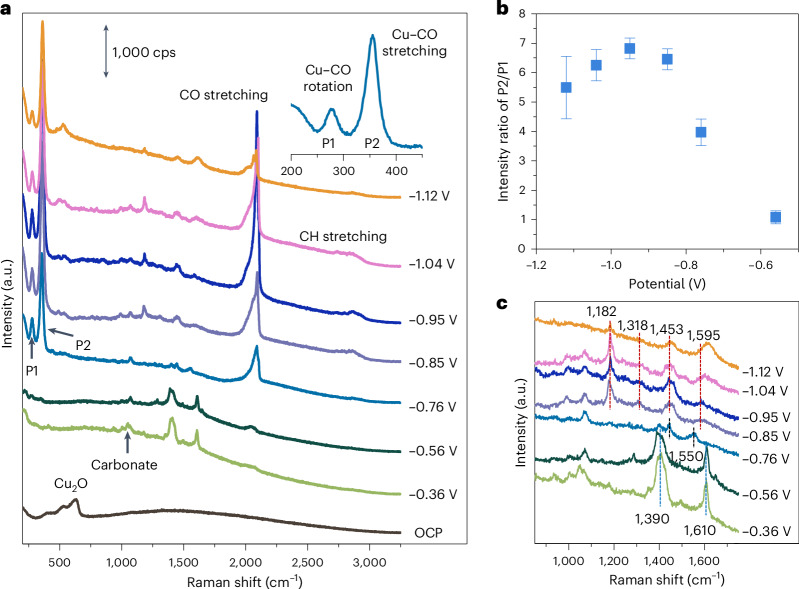
In situ SERS measurement during CO_2_RR. **a**, Raman spectra of an electrochemically treated Cu foil acquired during CO_2_RR for potentials ranging from the open-circuit potential (OCP) to about −1.1 V_RHE_ in a CO_2_-saturated 0.1 M NaClO_4_ electrolyte. cps, counts per second. **b**, Potential-dependent intensity ratio of P2 (Cu–CO stretching, ~360 cm^−1^) to P1 (restricted rotation of adsorbed CO, ~280 cm^−1^). **c**, Zoom-in Raman spectra in the region of 900–1,700 cm^−1^ from about −0.4 V_RHE_ to −1.1 V_RHE_, with relevant peaks highlighted via dashed lines. The error bars in **b** correspond to the s.d. of three independent measurements. Data are given as average ± s.d. Source data

To reveal molecular insights into C–C coupling intermediates and their potential dependence, we carried out in situ SERS measurements (Supplementary Fig. 7). Figure 2a shows the in situ Raman spectra acquired at the same position on the electrochemically treated Cu foil electrode as a function of the applied potential in a CO_2_-saturated 0.1 M NaClO_4_ electrolyte. A NaClO_4_ solution leading to a Raman band at ∼935 cm^−1^ (Supplementary Figs. 8 and 9) was used to decrease the influence of highly concentrated bicarbonate in the wavenumber range of 1,000–2,000 cm^−1^ (Supplementary Figs. 10 and 11). We note here that irrespective of the electrolyte anionic species, carbonate and bicarbonate ions will be present due to the CO_2_/H_2_O equilibrium. Although a lower Faradaic efficiency of C_2+_ products is obtained during CO_2_RR in CO_2_-saturated 0.1 M NaClO_4_ (ref. ^28^), the onset potentials and potential-dependent trends for ethylene and ethanol formation are similar to those in 0.1 M KHCO_3_ (Supplementary Fig. 12).

Initially, at open-circuit potential, Raman peaks at 400 cm^−1^, 528 cm^−1^ and 620 cm^−1^ from a CuO_*x*_ phase are detected^12,29^. At a potential of −0.36 V_RHE_, the CuO_*x*_ is reduced to metallic Cu on the surface, and a small peak is detected at 1,070 cm^−1^, typically assigned to carbonate^13^. Two new peaks at ∼1,390 cm^−1^ and ∼1,610 cm^−1^ appear, which are attributed to the symmetric and asymmetric stretching vibrations of carboxyl(ate) groups, respectively, based on control experiments in Ar-saturated NaClO_4_ with 1 mM HCOOH solution (Supplementary Fig. 13) and in Ar-saturated KHCO_3_ solution (Supplementary Fig. 11).

At −0.56 V_RHE_, peaks of CO adsorbed on Cu are detected, with vibrational features at ∼280 cm^−1^, 355–360 cm^−1^ and 1,970–2,110 cm^−1^ corresponding to the restricted rotation of adsorbed CO (P1), Cu–CO stretching (P2) and C–O stretching, respectively. Consistent with our recent finding on the CO coverage-driven C–C coupling mechanism^13^, the P2/P1 ratio, a proxy for CO surface coverage on copper, follows a volcano trend with the applied potential (Fig. 2b). At −0.76 V_RHE_, Raman bands at 490–530 cm^−1^ appear, which can be attributed to Cu–O stretching of oxygen or hydroxide species on Cu, or Cu–C stretching^12,29,30^. There are no indications of Cl^−^ related adsorbates, as the Raman band should appear at ∼300 cm^−1^ (refs. ^31,32^).

More interestingly, the peaks at 1,390 cm^−1^ and 1,610 cm^−1^ disappear with increasing CO surface coverage from −0.56 V_RHE_ to −0.76 V_RHE_, and two new peaks evolve at 1,450 cm^−1^ and 1,550 cm^−1^ (Fig. 2c), which were previously attributed to the *OCCO intermediate^15^. The observation of these peaks agrees well with a CO-coverage-determined C–C coupling during CO_2_RR^13^ and indicates a similar intermediate for C–C coupling during CORR and CO_2_RR. Concurrently, the Raman peaks of *HCOO^−^/*HCOOH disappear, which suggests that CO or C–C coupling intermediates may affect the production of formic acid due to competition for surface sites.

At more cathodic potentials, CO coverage further increases, and four new peaks can be detected at 1,182 cm^−1^, 1,318 cm^−^^1^, 1,453 cm^−^^1^ and 1,595 cm^−1^ from −0.85 V_RHE_, which show a regular shift with the potential, indicating the existence of new adsorbates. These peaks appeared simultaneously during the time-resolved in situ SERS experiment after a potential switch from −0.36 V_RHE_ to −0.96 V_RHE_ (Supplementary Fig. 14). Concurrently, a broad band in the range of 2,830–2,990 cm^−1^ appeared, which can be attributed to the stretching band of C–H (ref. ^12^).

## Intermediates responsible for CO_2_RR to ethylene and ethanol

To confirm the assignment of the vibrational modes detected experimentally, we reverted to DFT (PBE-D2^33^) through the Vienna Ab initio Simulation Package^34,35^. To mimic OD-Cu catalysts, we used two different models. Our first simulation cell consisted of a pristine OD-Cu structure, taken from a Cu(111)/Cu_2_O(111) epitaxy precursor optimized through ab initio molecular dynamics^36^. Due to the original oxygen-induced reconstruction, Cu surface sites with Cu–Cu coordination numbers varying from 4 to 10 were obtained (Supplementary Figs. 15 and 16). As a reference, a four-layer-thick (100) crystalline supercell (Supplementary Fig. 15) was used, as this facet is often deemed responsible for CO–CO dimerization^10^. Surface atoms in this system were characterized by a Cu–Cu coordination number of 8. To obtain the DFT vibrational frequencies of CO_2_RR reaction intermediates, we first optimized them on different active sites on the OD-Cu distorted surface and the crystalline Cu(100) and then carried out vibrational analysis on the final configurations. Simulated vibrational frequencies can be visualized through the ioChem-BD database^37^ (see the explanatory tutorial in Methods and Supplementary Fig. 17).

**Fig. 3:**
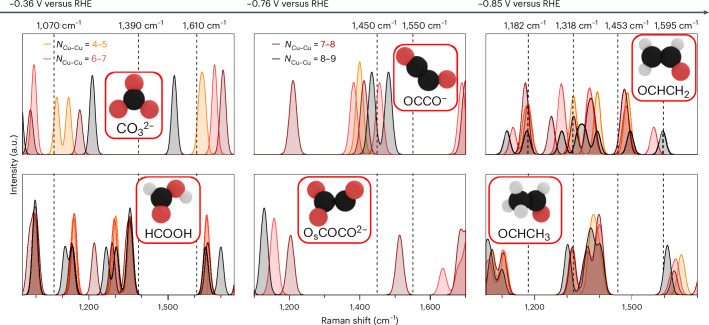
DFT-optimized CO_2_RR reaction intermediates and their vibrational fingerprints. DFT vibrational frequencies for *CO_3_^2−^, *COCO^−^, OCHCH_2_ (top, left to right), *HCOOH, *O_s_COCO^2−^ and *OCHCH_3_ (bottom, left to right) on adsorption sites with different coordination numbers, increasing from orange to black. Experimental signals at different applied potentials are indicated on top and highlighted in the panels by vertical dashed lines. Raman spectra were achieved by applying a smearing of 10 cm^−1^ on each DFT frequency and overlapping the resulting peaks. Inset: different CO_2_RR reaction intermediates, with H atoms given in white, O atoms in red and C atoms in black.

In Fig. 3, we show the main reaction intermediates responsible for the vibrational frequencies detected experimentally (Fig. 2a). After the reduction of the Cu oxidic phases, *CO_3_^2−^ and *HCOO^−^/*HCOOH species form (see 1,070 cm^−1^, 1,390 cm^−1^ and 1,610 cm^−1^ signals in Fig. 3; Supplementary Tables 1–3). Consistent with previous reports^14,15^, the signals appearing at 1,450 cm^−1^ and 1,550 cm^−1^ from −0.76 V_RHE_ can be attributed to *OCCO(H), with the C=O vibration mode (Fig. 3 and Supplementary Tables 4 and 5). Alternatively, such frequencies can be assigned to an OCCO^−^ dimer adsorbed on surface oxygen (O_s_), known as deprotonated glyoxylate O_s_COCO^2−^ (ref. ^36^), which also shows the 1,070 cm^−1^ band detected at −0.76 V_RHE_ (Supplementary Table 6).

Among other additional possible intermediates (*OCCHO, *OHCCOH, *OCCHOH, *CHO and *CH; Supplementary Tables 7–11), we attribute the 1,182 cm^−1^, 1,318 cm^−1^, 1,453 cm^−1^ and 1,595 cm^−1^ signals detected at −0.85 V_RHE_ and below to the CCO symmetric stretching, CCO antisymmetric stretching, C–C stretching and C–O (or C=C) stretching of OCHCH_2_ (or OCHCH_3_) species, respectively (Supplementary Table 7). Although this assignment is based on different independent experiments, we cannot fully rule out that the Raman bands observed might also result from (a set of) very similar adsorbates.

**Fig. 4:**
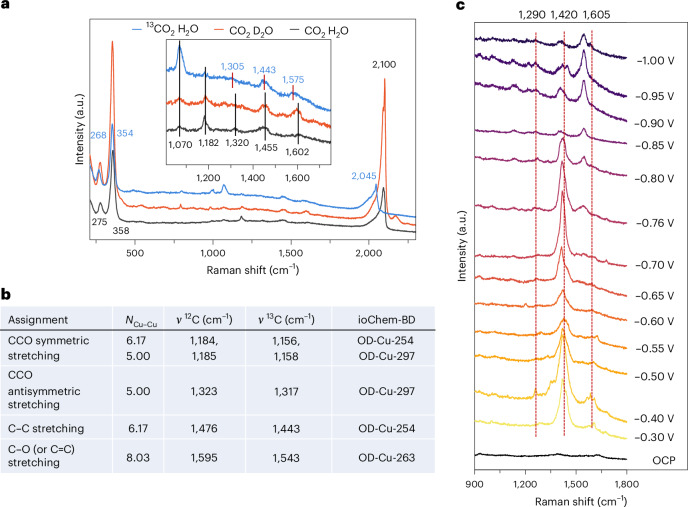
In situ SERS measurement and DFT vibrational analysis with the isotope exchange. **a**, Raman spectra of species adsorbed on the electrochemically treated Cu electrode at −0.95 V_RHE_ in ^13^CO_2_-saturated 0.1 M NaClO_4_ electrolyte and CO_2_-saturated 0.1 M NaClO_4_ D_2_O electrolytes. **b**, OCHCH_2_ vibrational fingerprints on selected active sites determined by DFT simulations for ^12^C and ^13^C (see Methods, ref. ^62^). **c**, Raman spectra of an electrochemically treated Cu foil acquired in 0.05 M glyoxal for potentials ranging from −0.3 V_RHE_ to about −1.0 V_RHE_ in an Ar-saturated 0.1 M NaClO_4_ electrolyte.

In addition, we carried out ^13^C- or D-labelling SERS experiments at −0.95 V_RHE_ (Fig. 4a). ^13^C/^12^C exchange led to obvious redshifts of the 1,455 cm^−1^ and 1,602 cm^−1^ peaks, in addition to the CO stretching (2,100 cm^−1^), Cu–CO stretching (358 cm^−1^) and CO rotation (275 cm^−1^) bands, proving that these peaks are related to the CO_2_RR process. Such redshifts were also confirmed by DFT vibrational analysis of O^12^CH^12^CH_2_ and O^13^CH^13^CH_2_ (Fig. 4b and Supplementary Table 12). Water shows a broad O–H bending band at about 1,600 cm^−1^ (refs. ^14,15^) (Supplementary Table 13), limiting the identification of vibrations from other species in this regime. In agreement with our assignment, the peak at about 1,602 cm^−1^ still appears after the D_2_O/H_2_O isotope exchange. Besides, the D_2_O/H_2_O exchange negligibly affected the peaks at about 1,320 cm^−1^ and 1,455 cm^−1^. For the peak at 1,182 cm^−1^, no obvious shift was observed in the D-labelling experiment, while the low intensity in the ^13^C/^12^C exchange experiment did not allow for a robust determination of the isotope-induced shift.

Since similar C–C coupling intermediates were proposed for CORR^3,38^, to validate our observations, we performed in situ SERS during CORR. Our data show similar bands (Supplementary Fig. 18). We further prove the appearance of C–C coupling intermediates by observing analogous signals on a different Cu foil with distinct surface morphology, yet with still good performance towards C_2+_ products (Supplementary Fig. 19).

As ethanol and acetaldehyde are the main C_2_ products for glyoxal reduction on polycrystalline copper and are proposed to share the same *OCHCH_2_ intermediate^16–18^, we performed in situ Raman experiments of glyoxal and ethanol reduction (Supplementary Figs. 20 and 21). As shown in Fig. 4c, similar bands at about 1,290 cm^−1^, 1,420 cm^−1^ and 1,605 cm^−1^ are observed during glyoxal reduction. We attribute the variations in relative intensities and positions to the substantially different chemical environment in the absence of predominantly co-adsorbed CO, as is the case in CO_2_RR. The increasing relative intensity of the band at ∼1,550 cm^−1^ is probably caused by key intermediates for ethylene glycol formation, which is the dominant product of glyoxal reduction at lower potentials^20^. Instead, these signals were not observed for ethanol reduction (Supplementary Fig. 21). Thus, we reasonably confirmed the detection of a common intermediate along the CO, CO_2_ and glyoxal reduction route to ethanol, which is *OCHCH_2_. Overall, the presence of CO molecules on the Cu surface during CO_2_RR is linked to and probably causes the formation of *CO–CO or *CO–COH at about −0.76 V_RHE_, and then these species further reduce to, for example, *OCHCH_2_ at −0.85 V_RHE_ and −0.95 V_RHE_.

## Cu active sites selective for CO_2_RR to ethylene and ethanol

**Fig. 5:**
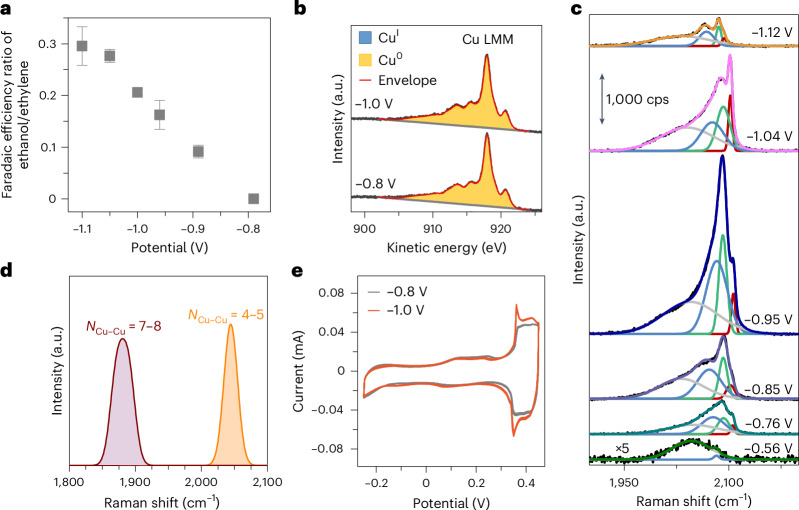
Defect-driven mechanisms for CO_2_RR to ethylene and ethanol. **a**, Ratio of the Faradaic efficiencies of ethanol and ethylene as a function of the applied potential. **b**, Quasi in situ Cu LMM XAES spectra of electrochemically pre-treated Cu foils after 1 h of CO_2_RR at −0.8 V_RHE_ and −1.0 V_RHE_ without air exposure. **c**, Dependence of the C–O stretching band on the applied potential. The Raman spectra are fitted with the sum of 4 Gaussian functions with peaks at about 2,035 cm^−1^ (grey), 2,075 cm^−1^ (blue), 2,090 cm^−1^ (green) and 2,100 cm^−1^ (red). The Raman data in **c** are from the same dataset as those in Fig. 2. **d**, DFT C–O stretching wavenumber as a function of the Cu–Cu coordination number, decreasing from orange (*N*_Cu–Cu_ = 4–5) to dark brown (*N*_Cu–Cu_ = 7–8). **e**, Cyclic voltammograms of the electrochemically treated Cu foil after 1 h of CO_2_RR at −0.8 V_RHE_ and −1.0 V_RHE_. Cyclic voltammetry was performed in an Ar-saturated 0.1 M NaOH with a scan rate of 50 mV s^−1^. The error bars in **a** correspond to the s.d. of three independent measurements. Data are given as average ± s.d. Source data

The potential-dependent formation of C–C coupling intermediates explains the selectivity trend towards C_2+_ products observed during CO_2_RR. In correspondence with an increase in the CO coverage from −0.56 V_RHE_ to −0.76 V_RHE_, the *OCCO intermediate forms on the surface and evolves to ethylene at −0.76 V_RHE_. Instead, the *OCHCH_*x*_ key intermediate is detected from −0.85 V_RHE_, the observed onset potential for CO_2_RR to alcohols. Besides, the ratio of Faradaic efficiencies towards ethanol and ethylene continuously increases with increasingly negative applied potential (Fig. 5a).

To further correlate the catalyst structure, surface intermediates and reaction products, we carried out quasi in situ Cu LMM XAES, in situ SERS, cyclic voltammetry and simulations. The surface of the electrochemically treated Cu foil was fully reduced to metallic Cu after CO_2_RR at −0.8 V_RHE_ (Fig. 5b). Figure 5c shows the potential-dependent variations of the C–O stretching band of adsorbed CO during CO_2_RR. The C–O stretching frequency of atop CO on Cu depends on the coordination of the underlying metal atom^39,40^; thus, it can be used as a probe to track the potential-dependent density of different active sites. In principle, the peak shifts to higher wavenumbers for adsorption sites with lower coordination number (although other effects, such as dipole coupling, can interfere)^40^, consistent with DFT simulations (Fig. 5d and Supplementary Table 14). Four Gaussian peak functions centred at about 2,035 cm^−1^, 2,075 cm^−1^, 2,090 cm^−1^ and 2,100 cm^−1^ were used to fit the recorded potential-dependent Raman spectra. C–O stretching bands with intermediate frequencies (2,075 cm^−1^ and 2,090 cm^−1^) become the main contribution at −0.76 V_RHE_, from which the *OCCO intermediate is identified and ethylene is produced, in line with the observation of a 2,060 cm^−1^ CO precursor reacting towards C–C coupling as reported in a previous SERS study^6^. At −0.95 V_RHE_, the C–O stretching bands at 2,090 cm^−1^ and 2,100 cm^−1^, assigned to atop CO adsorbed on defect Cu sites, further increase and reach a maximum at −1.04 V_RHE_, coinciding with the detection of the *OCHCH_*x*_ key intermediate and ethanol.

The formation of defects on the Cu surface during CO_2_RR, for instance, adatoms or small Cu clusters with low-coordinated Cu atoms, has been experimentally observed via electrochemical scanning tunnelling microscopy^41^. Moreover, the subsurface incorporation of atomic impurities such as C or O during CO_2_RR cannot be ruled out, as this could also lead to a defective Cu surface with the modified Raman shifts obtained here. Cyclic voltammetry has been used to compare the defect density on the Cu surface after CO_2_RR using the charge transferred above +0.3 V_RHE_ (ref. ^25^). Clearly, CO_2_RR at −1.0 V_RHE_ induced a higher defect density compared with −0.8 V_RHE_ (Fig. 5e), and the cyclic voltammogram agrees well with that of a restructured/roughened Cu foil upon pulsed electrolysis into the Cu^2+^ regime^25^. These findings are further supported by Pb underpotential deposition experiments^42,43^, during which an increased defect site density of CO_2_RR at −1.0 V_RHE_ was observed (Supplementary Fig. 22). Finally, adsorbed CO linked to a lower C–O stretching band Raman shift becomes preeminent beyond −1.1 V_RHE_, where CH_4_ production again significantly increases, in line with a previous report^44^. Thus, both in situ SERS and cyclic voltammetry hint at the key role of structural defects in tuning the ethylene/ethanol ratio. These defects are probably formed under reaction conditions and generate distinct surface sites with strong CO binding.

On the basis of these mechanistic insights, we aimed to correlate specific active sites and reaction intermediates with ethylene and ethanol production during CO_2_RR. Recent experimental studies have highlighted the relevance of defects and polarized Cu sites in enabling CO_2_RR^25,26,45,46^, while flat atomically ordered surfaces mainly yield H_2_ (ref. ^24^). Moreover, defective structures and highly disordered Cu(0)/Cu(I,II) interfaces created through pulsed electrolysis conditions were found to significantly enhance ethanol selectivity^25,45,46^. Thus, we initially assessed the role of two structural descriptors to identify defects among the considered active sites: their coordination number (*N*_Cu–Cu_; Supplementary Fig. 23) and the relative compressive three-dimensional strain, calculated by dividing the distances between the active site and neighbouring atoms by the Cu–Cu bulk distance (Σ_*i*_(Δ*d*_*i*_/*d*)*N*_*i*,Cu–Cu_^−1^ with *i* ranging from 1 to the number of neighboring atoms, see Supplementary Table 15 and Methods for additional details). Remarkably, the strain does not scale with the coordination number for the distorted sites from the OD-Cu model. Instead, on crystalline domains such as Cu facets and adatoms, both parameters correlate (Supplementary Fig. 23), suggesting a mutual dependence of coordination- and strain-based linear scaling relationships for crystalline sites^47,48^.

To sample the reactivity of such surface defects, we estimated the binding energy of atop *CO and *OCHCH_2_ versus *N*_Cu–Cu_ and Σ(Δ*d*/*d*)*N*_Cu–Cu_^−1^. *CO was taken as a C_2+_ selectivity descriptor^10^, whereas *OCHCH_2_ binding strength was used as a descriptor of OCHCH_2_ stability on the surface. On crystalline domains, *CO binding depends weakly on the strain, in line with results on crystalline surfaces^47^, while differences of more than 0.4 eV are observed on highly strained low-coordinated OD-Cu sites (Supplementary Fig. 24 and Supplementary Table 16). Besides, both crystalline and distorted morphologies with coordination number below 8 show strong CO binding (<−0.2 eV).

**Fig. 6:**
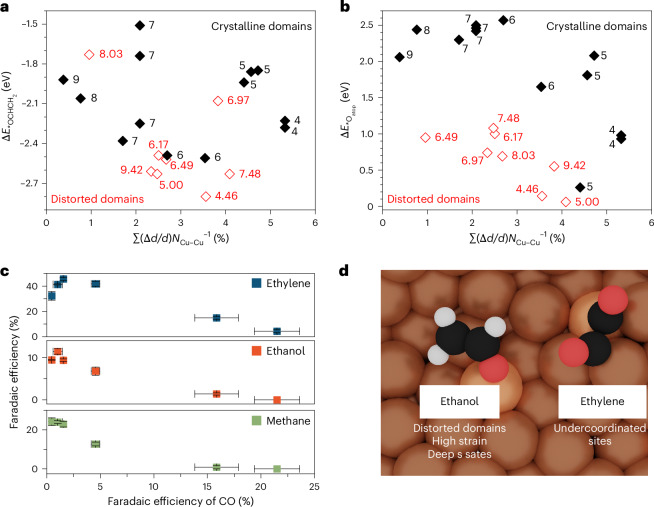
Morphological descriptors for CO_2_R to C_2+_ products. **a**,**b**, Correlation of *OCHCH_2_ (ΔE_*OCHCH2_) (**a**) and *O atop (ΔE_*Oatop_) binding energies (**b**) versus Σ(Δ*d*/*d*)*N*_Cu–Cu_^−1^. Cu–Cu coordination numbers are reported as labels. Red data points indicate Cu sites on distorted domains, and black data points represent crystalline domains. **c**, Comparison between ethylene, ethanol and methane Faradaic efficiency versus CO Faradaic efficiency. Data retrieved from Fig. 1f. **d**, Key reaction intermediates and properties of the active sites selective towards ethanol and ethylene, with H atoms given in white, O atoms in red and C atoms in black. The error bars in **c** correspond to the s.d. of three independent measurements. Data are given as average ± s.d. Source data

Localized compressive strain plays a crucial role in stabilizing *OCHCH_2_ species on distorted sites (red empty data points in Fig. 6a). In fact, *OCHCH_2_ formation energies increase following the increment of compressive strain, and stronger binding is observed on undercoordinated distorted sites rather than crystalline sites. Such difference between crystalline and distorted sites can be ascribed to their *s*-band states (Supplementary Table 16). Distorted domains present deeper *s*-band states, whereas crystalline sites are characterized by positive *s*-band centres (versus the Fermi energy). The *s*-band equally affects a previously employed descriptor for ethylene/ethanol competition, the *O binding strength^49^. Crystalline sites with a positive *s*-band centre show weak *O binding (black data points in Fig. 6b), whereas distorted sites with a negative *s*-band centre enable strong *O binding, thus promoting the adsorption of intermediates with terminal oxygen along the ethanol route^49^. The *s*-band centre shows a strong correlation with the *d*-band centre (Supplementary Fig. 25 and Supplementary Table 17). Finally, we observe that scaling relationships between the binding energy of adsorbed molecules and strain are heavily affected by the nature of active sites (Supplementary Note 2).

Overall, these insights highlight a clear correlation between active sites and product distribution. Undercoordinated Cu sites enable strong *CO binding, thus sustaining a high CO coverage on the surface, which opens the ethylene pathway via an OCCO(H) intermediate, explaining the evidence of C_2_H_4_ as the only C_2+_ product observed at −0.76 V_RHE_ (Fig. 1f). This scheme explains the typically high CO_2_RR selectivity towards C_2_ of OD-Cu^25^, which is due to the abundant undercoordinated sites on these materials^36^ (about 74% for *N*_Cu–Cu_ < 8 on our OD-Cu model; Supplementary Fig. 16). Furthermore, from −0.85 V_RHE_ onwards, undercoordinated distorted sites with high surface strain and deep *s*-band states originate (Fig. 5c,e) and enable the formation of OCHCH_2_ at the surface (Fig. 6b), detected experimentally through Raman spectroscopy (Figs. 2c and 3) together with the production of ethanol (Fig. 1f).

A careful comparison of ethylene, ethanol and methane Faradaic efficiency versus CO selectivity (taken from Fig. 1f) confirms the existence of key differences between the ethylene and ethanol routes (Fig. 6c). Instead, the similar dependence of ethanol and methane Faradaic efficiency on CO selectivity supports the hypothesis of CH_*x*_ as common precursors for the two products, providing additional parallel pathways from CO–CH_*x*_ coupling, in line with the recent CH_3_I/CO reduction study^20^. These undercoordinated distorted sites open the OCHCH_2_-mediated ethanol route (Fig. 6d).

## Proposed reaction scheme for CO_2_RR to C_2+_ products

**Fig. 7:**
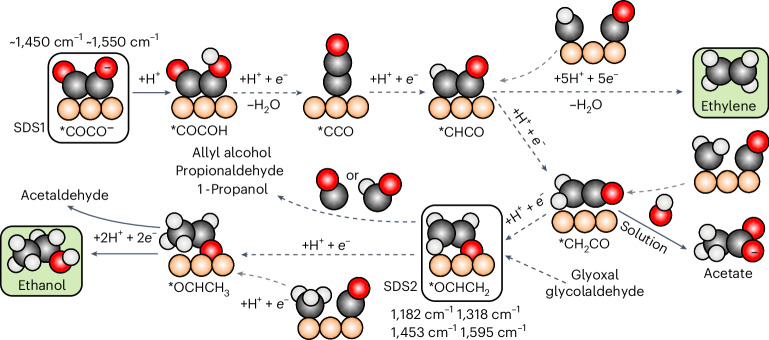
Proposed reaction scheme for CO_2_ reduction to C_2+_ products. Proposed reaction scheme, including ethylene and ethanol, was extracted from the present experimental Raman data, our DFT data and additional mechanistic insights reflected in refs. ^16,21,51,63^. The main C_2+_ products are highlighted in green. Reaction steps confirmed by experimental studies are reported as solid lines, and proposed steps are labelled as dashed lines. Grey arrows indicate potential CO–CH_*x*_ coupling routes. H atoms are given in white, O atoms in red and C atoms in black.

The proposed OCHCH_2_-mediated ethanol pathway suggests an updated reaction scheme for CO_2_RR towards C_2+_ products (Fig. 7). Ethylene and ethanol formations are pH-independent on the RHE scale^5^. Thus, according to the state of the art^2,7^, CO–CO dimerization, which involves a single electron transfer, is assumed to be the rate-determing step (RDS) towards multi-carbon molecules during CO_2_ reduction. Such hypothesis has been recently challenged by mechanistic studies of CO reduction^50^; thus, competing RDSs may exist depending on the catalyst morphology. If formed, the CO–CO(H) species is characterized by either a single or double C–C bond^7^ (Supplementary Table 18) and acts as a selectivity switch between C_1_ and C_2+_ products (C_2+_ selectivity-determining step 1, SDS1). The hypothesis of OCCO(H) as a common C_2+_ precursor for CO_2_ reduction is reinforced by the strong correlation between ethanol and ethylene Faradaic efficiency on copper (*R*^2^ = 0.90, values taken from ref. ^51^; see Supplementary Fig. 26 and Supplementary Table 19). In line with recent advances^20^, additional parallel pathways, such as CO–CH_*x*_ coupling (grey arrows, Fig. 7), may also open, yet our DFT simulations disprove any major role of these on distorted sites. In fact, the protonation of CH_*x*_ to CH_*x*+1_ species (and eventually to methane) is always more favourable than the CO–CH_*x*_ coupling, apart from the CO–CH case (Supplementary Fig. 27).

Once formed, CO–CO(H) dimers quickly protonate and evolve into an adsorbed ketene *CCO upon the loss of one H_2_O molecule, as previously suggested^21,27^. Such ketene then reduces to *CHCO upon proton-coupled electron transfer^7,21^, the last common precursor between ethylene and ethanol^49^. *CHCO can then either reduce to *OCCH_2_ along the acetate/ethanol pathway or undergo reduction to ethylene via five proton-coupled electron transfers and the release of one H_2_O molecule^21^. Along the acetate/ethanol route, OCCH_2_ can desorb and react in solution with OH^−^ to form acetate^21^. Following the C_2_ route, when highly strained distorted sites are present, an alternative reaction pathway opens, that is, enabling the reduction of *OCCH_2_ to *OCHCH_2_ by strongly binding the terminal oxygen in a monodentate configuration. *OCHCH_2_ (ref. ^7^; characterized by a double C–C bond, see Supplementary Table 18) can either reduce to acetaldehyde and ethanol or couple with a CO/COH species to form allyl alcohol and, at later reduction stages, 1-propanol^51^ (see the correlation between Faradaic efficiency for both products in Supplementary Fig. 26 and Supplementary Table 19).

Thus, although a methyl carbonyl intermediate (OCCH_3_) was recently suggested as the ethanol precursor during CH_3_I and acetaldehyde/CO reduction^20^, we here identify *CHCO reduction to *OCHCH_2_ as the selectivity switch (selectivity-determing step 2, SDS2) to ethanol, acetaldehyde, 1-propanol and allyl alcohol (Supplementary Note 1 and Supplementary Figs. 28 and 29), as confirmed by the strong correlation between acetaldehyde and ethanol selectivities (*R*^2^ = 0.82 (ref. ^51^); Supplementary Fig. 26 and Supplementary Table 19). Besides, in situ Raman spectroscopy during CO_2_, CO and glyoxal reduction to ethanol confirms the existence of a shared reaction intermediate, which is reasonably OCHCH_2_ (Supplementary Fig. 28). The reduction of OCHCH_2_ to OCHCH_3_ is thermodynamically sluggish (Supplementary Fig. 30), which might explain the experimental observation of OCHCH_*x*_ species, reflecting a significant population on the Cu surface to be detected via SERS on a very rough Cu surface. In fact, a strong *OCH_2_CH_3_ signal was also observed on other ethanol-selective catalysts^52^.

## Conclusion

On the basis of in situ SERS and DFT simulations, we identified the crucial active sites and reaction intermediates responsible for ethylene and ethanol formation in CO_2_RR over a Cu surface. We unveiled that defects in Cu with a low coordination number (<8) enable strong *CO binding, thus sustaining high surface CO coverages and subsequent CO–CO dimerization. These undercoordinated sites are mainly selective to ethylene via the traditional route, *CO–CO or *CO–COH couplings, which are C_2+_ product precursors. However, defects characterized by a highly compressed and distorted coordination environment and deep *s*-band states are instead generated at higher overpotentials and stabilize the adsorption of OCHCH_2_ via the terminal oxygen, opening an alternative selective route towards ethanol on copper. By combining the potential-dependent evolution of CO adsorption and C–C coupling intermediates, we here describe an updated CO_2_RR-to-C_2+_ reaction scheme that correlates between active sites, surface intermediates and products. Insights from the identified active sites provide key guidelines to rationally design electrocatalysts to maximize the selectivity of each of these products independently.

## Methods

### Electrode preparations

A commercial polycrystalline copper foil (Advent Research Materials, 99.995%) was used in all experiments. Before each experiment, the copper foil was electropolished at 3 V versus a titanium foil for 3 min in a H_3_PO_4_/H_2_SO_4_ solution consisting of 130 ml H_3_PO_4_ (VWR, 85 wt%), 20 ml H_2_SO_4_ (VWR, 95%) and 60 ml ultrapure water. After that, all working electrodes were rinsed with ultrapure water and placed in the electrochemical cell for the electrochemical oxidation–reduction process. The three-electrode system consisted of the copper foil as the working electrode, a platinum wire as the counter electrode and a leak-free Ag/AgCl reference electrode (LF-1, Alvatek). The copper electrodes were treated at room temperature by cyclic voltammetry in gas-saturated 0.1 M NaClO_4_ (Aldrich, 99.99%) without the use of halide, at a sweep rate of 10 mV s^−1^ from −1.3 V_RHE_ to +0.9 V_RHE_ for two cycles.

### Characterization of samples

The crystal structure of the catalysts was studied using XRD with a Bruker-AXS D8 Advance in parallel beam configuration equipped with a Cu X-ray tube, a Goebel mirror and equatorial Soller slit (0.3°) as well as an energy-dispersive LynxEye detector. The diffraction pattern was recorded through the grazing incidence mode with an incidence angle of 1° to decrease the bulk contribution of the Cu foil.

The size and morphology of the samples were determined by SEM with a ThermoFisher Apreo microscope and a Hitachi S-4800 system, including a cold field emission gun.

XPS measurements were performed with a commercial hemispherical analyser (Phoibos 150, MCD-9 Detector, SPECS, *E*_pass_ = 15 eV) and a monochromatic X-ray source (SPECS) with an Al anode (*E*_Kα_ = 1,486.7 eV as X-ray energy) at a power of *P* = 300 W. All spectra were aligned by fixing the Cu 2*p*_3/2_ of Cu^0^ and Cu^+^ to 932.67 eV as a reference.

### In situ Raman experiments

The in situ Raman spectra were obtained using a Renishaw (InVia Reflex) confocal Raman microscope with a 785 nm laser. A water immersion objective with a long working distance (Leica Microsystems, ×63, numerical aperture of 0.9) was chosen to perform the in situ experiments in an electrolyte. The objective with a long working distance was needed to avoid diffusion hindrance during the Raman measurements^13^. During the measurements, the objective was protected from the electrolyte by a Teflon film (DuPont, film thickness of 0.013 mm). The laser power was about 0.36 mW. The acquisition time was 10 s for the steady-state experiments at different potentials. The spot size of the focused laser beam was about 2 μm in diameter. The electrochemical measurements were performed in a home-built spectro-electrochemical cell made of Teflon and controlled by a Biologic SP-240 potentiostat. The cell was equipped with a reference electrode (leak-free Ag/AgCl, Alvatek), a counter electrode (Pt ring) and a Cu foil working electrode. Typically, a 15 ml CO_2_-saturated 0.1 M NaClO_4_ solution was used as the electrolyte, and CO_2_ was continuously injected into the solution during the experiment. Ar-saturated 0.1 M KHCO_3_ and Ar-saturated 0.1 M NaClO_4_ were used in the experiments as well. For the steady-state experiment, each potential was applied for at least 10 min before collecting the spectra to ensure steady-state conditions at the surface of the catalyst.

### CO_2_RR testing

Electrocatalytic measurements were performed with a Biologic SP-240 potentiostat in an H-type cell equipped with an anion exchange membrane (Selemion AMV, AGC). The three-electrode system consisted of the Cu foil as the working electrode, a platinum gauze (MaTecK, 3,600 mesh cm^−2^) as the counter electrode and a leak-free Ag/AgCl reference electrode (LF-1, Alvatek). A CO_2_-saturated 0.1 M KHCO_3_ aqueous solution, purified from trace metal ion impurities by a cation-exchange resin (Chelex 100 Resin, Bio-Rad), was used as the electrolyte. The CO_2_ (99.995%) flow rate was 20 ml min^−1^. For each cathodic potential, the chronoamperometry lasted 3,600 s. All potentials are given versus the RHE scale and were corrected for the *iR* drop. Each presented data point corresponds to an identical freshly prepared sample following this protocol at different potentials. The electrochemical surface roughness factor, estimated from double-layer capacitance measurements, is 14.6, which was measured by cyclic voltammetry in a non-Faradaic potential range from 0.10 V_RHE_ to 0.25 V_RHE_ at scan rates of 20 mV s^−1^, 40 mV s^−1^, 60 mV s^−1^, 80 mV s^−1^ and 100 mV s^−1^ in a CO_2_-saturated 0.1 M KHCO_3_ solution after 1 h of electrochemical reaction.

Gas products were detected and quantified every 15 min by online gas chromatography (Agilent 7890B), equipped with a thermal conductivity detector and a flame ionization detector. Liquid products were analysed after each measurement with a high-performance liquid chromatograph (Shimadzu Prominence), equipped with a NUCLEOGEL SUGAR 810 column and a refractive index detector, and a liquid gas chromatograph (Shimadzu 2010 Plus), equipped with a fused silica capillary column and a flame ionization detector^13^.

All catalytic results in this study are shown in terms of Faradaic efficiency. The Faradaic efficiency of the gas product *x* was calculated using equation (1):

1 $${\mathrm{FE}}=\frac{\dot{V}\,\times {C}_{x}\,\times \,{z}_{x}\,\times \,\mathrm{F}}{A\,\times {V}_{{\mathrm{M}}}\,\times {j}_{{\mathrm{total}}}}\times 100 \% ,$$

and for the liquid product *x* was calculated using equation (2):

2 $${\mathrm{FE}}=\frac{V\times {\Delta C}_{x}\times {z}_{x}\times \mathrm{F}}{\Delta Q}\times 100 \% .$$

Here FE is the Faradaic efficiency of product *x*, $\dot{V}$ is the CO_2_ gas flow rate (l s^−1^), *C*_*x*_ is the volume fraction of the product *x* detected by gas chromatography, *z*_*x*_ is the number of electrons transferred for reduction to product *x*, *F* is the Faradaic constant (C mol^−1^), *A* is the geometric area of the electrode (cm^−2^), *V*_M_ is the molar volume (22.4 l mol^−1^), *j*_total_ is the total current density during CO_2_ bulk electrolysis (A cm^−2^), Δ*C*_*x*_ is the final concentration of product detected by high-performance liquid chromatography and liquid gas chromatography (mol l^−1^), Δ*Q* is the total charge transferred during electrolysis at constant potential or current (C), and *V* is the volume of the electrolyte (l).

### Quasi in situ XPS

Quasi in situ XPS experiments were performed to avoid exposure of the sample to air after the electrochemical treatment. In this set-up, an electrochemical cell is directly attached to the UHV system where the XPS chamber is located to allow the sample transfer without air exposure. The electrochemical measurements were carried out using a potentiostat (Autolab PGSTAT 302N) and a CO_2_-saturated 0.1 M KHCO_3_ electrolyte. After the electrochemical treatment, the samples were rinsed with Ar-saturated H_2_O and subsequently transferred under an Ar atmosphere into the load-lock of the UHV system.

### DFT details

The DFT calculations were performed using the Vienna Ab initio Simulation Package^34,35^, with the Perdew-Burke-Ernzerhof (PBE) density functional^33^. We included dispersion according to the DFT-D2 method^53,54^, with the re-parametrization of *C*_6_ coefficients for metals performed by our group^55^. To account for solvent stabilization of intermediate formation energy, we employed the VASPsol code^56,57^. Inner electrons were represented by projector augmented wave (PAW) pseudopotentials^58,59^, whereas the monoelectronic states for the valence electrons were expanded as plane waves with a kinetic energy cut-off of 450 eV.

We modelled the OD-Cu catalyst investigated experimentally by considering a Cu(111)/Cu_2_O(111) epitaxy with surface reconstruction induced through ab initio molecular dynamics for 1 + 10 ps (3 fs time step)^36^, and a 4-layer thick crystalline Cu(100). For the Cu(100), the two uppermost layers were fully relaxed and the rest fixed to the bulk distances. The Brillouin zone was sampled by a Γ-centred *k*-points mesh from the Monkhorst–Pack method^60^, with a reciprocal grid size smaller than 0.03 Å^−1^. The vacuum between the slabs was larger than 12 Å. The adsorbates were placed only on one side of the slab, thus requiring a dipole correction to remove spurious contributions arising from the asymmetric configuration^61^. Formation energies for the selected intermediates were obtained assuming CO_2_(g), H_2_(g), H_2_O(g) and clean surfaces as energy references.

### Cu–Cu coordination number

Two Cu atoms are coordinated if their distance falls below a certain threshold. Since bulk Cu (face-centred cubic) and Cu_2_O show Cu–Cu bond distances of 2.57 Å and 3.05 Å, respectively, we assigned a coordination number *N*_Cu–Cu_ for metallic Cu between 0.0 (*d*_Cu–Cu_ = 3.05 Å) and 1.0 (*d*_Cu–Cu_ = 2.57 Å) following a decay controlled by the error function (erf)^36^, as shown in equations (3) and (4). In equation (3), *N*_Cu–Cu_ is the Cu–Cu coordination number (dimensionless), and *d*_Cu–Cu_ is the Cu–Cu distance (Å). In equation (4), *t* (dimensionless) is the variable of integration, and 0 and *z* represent the integration limits:

3 $${N}_{{\mathrm{Cu}}-{\mathrm{Cu}}}=\frac{1}{2}-\frac{1}{2}{\mathrm{erf}}\left(\frac{{d}_{{\mathrm{Cu}}-{\mathrm{Cu}}}-2.57{\text{\AA }}}{0.1{\text{\AA }}}\right)$$

4 $${\mathrm{erf}}\left(z\right)=\frac{2}{\sqrt{{\mathrm{\pi }}}}{\int_{0}^{{\mathrm{z}}}}\exp \left(-{t}^{2}\right){{\mathrm{d}}t}$$

### Three-dimensional localized strain

Localized three-dimensional strain for a given Cu active site *j* was estimated by assessing the local deformation of Cu–Cu distances versus the bulk value (*d*_Cu–Cu_(bulk) = 2.57 Å; Supplementary Table 15) for a given atom *i* within the coordination shell of species *j*, as shown in equation (5). Then, such deformations were summed among all the neighbouring Cu atoms and normalized by the Cu–Cu coordination number (*N*_Cu–Cu_) of the active site *j*, as shown in equation (6). Distances are given in Å, coordination numbers are dimensionless, and three-dimensional strain is reported as a percentage of deformation. Negative values of strain indicate expansion, whereas positive values characterize contraction:

5 $$3{\mathrm{D}}\; {\mathrm{strain}}(j,i)=\frac{{d}_{{\mathrm{Cu}}-{\mathrm{Cu}}}\left({\mathrm{bulk}}\right)-{d}_{{\mathrm{Cu}}-{\mathrm{Cu}}}\left(j,i\right)}{{d}_{{\mathrm{Cu}}-{\mathrm{Cu}}}\left({\mathrm{bulk}}\right)}\times 100$$

6 $$\Sigma (\Delta d/d){N}_{{\mathrm{Cu}}-{\mathrm{Cu}}}^{-1}(j)=\mathop{\sum }\limits_{i=1}^{{N}_{{\mathrm{Cu}}-{\mathrm{Cu}}}}\frac{3{\mathrm{D}}\; {\mathrm{strain}}(j,i)}{{N}_{{\mathrm{Cu}}-{\mathrm{Cu}}}^{-1}(j)}$$

### Visualization of vibrational fingerprints

To visualize vibrational frequencies of a given reaction intermediate in ioChem-BD^37^, it is necessary to follow the next steps, summarized in Supplementary Fig. 17.

1. Access ref. ^62^ and click on the ‘frequencies’ collection (top left)
2. Select the desired item to visualize by clicking on the title ‘frq-X’
3. Once a new tab opens, click on ‘Actions → View data’ (top right)
4. Once a new tab opens, scroll down to ‘vibrational frequencies’ and click on it
5. Click on ‘Load vibrations’
6. Rotate the point of view by pressing the left button of the mouse and select the desired frequency

## Supplementary information

Supplementary Information Supplementary Notes 1 and 2, Figs. 1–30, Tables 1–19 and References.

Supplementary Data 1 Source data for Supplementary Fig. 12.

## Source data

Source Data Fig. 1 Statistical source data for Fig. 1e,f.

Source Data Fig. 2 Statistical source data for Fig. 2b.

Source Data Fig. 5 Statistical source data for Fig. 5a.

Source Data Fig. 6 Statistical source data for Fig. 6c.

## Data Availability

Regarding the experimental datasets, the data are available in the main text, in the Supplementary Information file or as Source Data files for Figs. 1e,f, 2b, 5a, 6c and Supplementary Fig. 12. Additional data are available from the corresponding authors upon reasonable request. The DFT datasets generated during the current study are available in the ioChem-BD database^37^ at 10.19061/iochem-bd-1-251 (ref. ^62^).
