# Supplementary material for: Key intermediates and Cu active sites for CO2 electroreduction to ethylene and ethanol
Source: Nat Energy. 2024 Sep 11;9(12):1485–96. doi: 10.1038/s41560-024-01633-4 (PMC11659170; doi:10.1038/s41560-024-01633-4)
Supplement: Supplementary file 1 — Supplementary Notes 1 and 2, Figs. 1–30, Tables 1–19 and References. [file 41560_2024_1633_MOESM1_ESM.pdf]

nature energy

Supplementary information https://doi.org/10.1038/s41560-024-01633-4

Key intermediates and Cu active sites for CO_2 electroreduction to ethylene and ethanol

In the format provided by the authors and unedited

The PDF file includes:

Supplementary Notes 1,2

Supplementary Figures 1-30

Supplementary Tables 1-19

Supplementary References

Supplementary Notes

Supplementary Note 1 | Identification of $*OCHCH_2$ formation as selectivity-determining step toward ethanol.

According to Ref.[1], only acetaldehyde and ethanol were observed by Online Electrochemical Mass Spectrometry (OLEMS) during reduction of 0.05 M glyoxal and 0.05 M glycolaldehyde on copper in a phosphate buffer (0.1 M K_2HPO_4 + 0.1 M KH_2PO_4, pH 7). Successive mechanistic studies on glyoxal reduction demonstrated that such intermediate mainly reduces to ethanol and ethylene glycol, with acetaldehyde and glycolaldehyde as minor reaction products. [1–3] Ethylene almost does not appear during glyoxal reduction, with partial current density around 0.01 mA/cm^{-2} at –1.0 V vs RHE for 0.02 mol l^{-1} glyoxal in 0.1 mol l^{-1} phosphate buffer.

Typically, the absence of ethylene and the concurrent formation of ethylene glycol at significant rates during glyoxal reduction (opposite to the low rates reported during CO_2 reduction)[4] have been assumed as strong proofs that glyoxal is not an intermediate during CO_2 reduction to C_{2+} products. In line with Ref.[5], we here suggest that glyoxal and glycolaldehyde reduce to ethylene glycol through the $*OCHCH_2OH$ intermediate, which (1) either converts to $*OCHCH_2$ through one proton-coupled electron (PCET) step and the loss of a water molecule, or (2) to ethylene glycol $(CH_2OH)_2$ via two PCET steps (Supplementary Figure 28). While $*OCHCH_2OH$ and ethylene glycol are not along the CO_2 reduction pathway, motivating the low formation rates observed during CO_2RR, we propose that $*OCHCH_2$ is the crucial exclusive precursor for acetaldehyde, ethanol, and 1-propanol. Further, due to the observed absence of ethylene during glyoxal/glycolaldehyde reduction, we propose that $*OCHCH_2$ is not an ethylene precursor, contrarily to the current state-of-the-art assumption.[6]

Supplementary Note 2 | Effect of active sites' morphology on scaling relationships between strain and binding energy

We here observe that previous studies on strained crystalline surfaces reported an opposite trend than the one reported in Fig. 6a-b, Supplementary Figure 24, i.e. stronger binding energies at higher expansive strain and lower binding elsewhere.[7,8] Such different behavior is ascribable to the distinct morphologies of the selected sites, respectively locally distorted Cu clusters in our case and crystalline surface with varying lattice constant in Refs.[7,8]. While the expansion of the lattice constant leads to an upshift of the d-band center for crystalline domains, such trends is reverted for the distorted sites here modeled (see Supplementary Table 15). Such evidence confirms the need for further mechanistic studies on distorted domains, which have completely different properties than the crystalline well-ordered surfaces.

Supplementary Figures

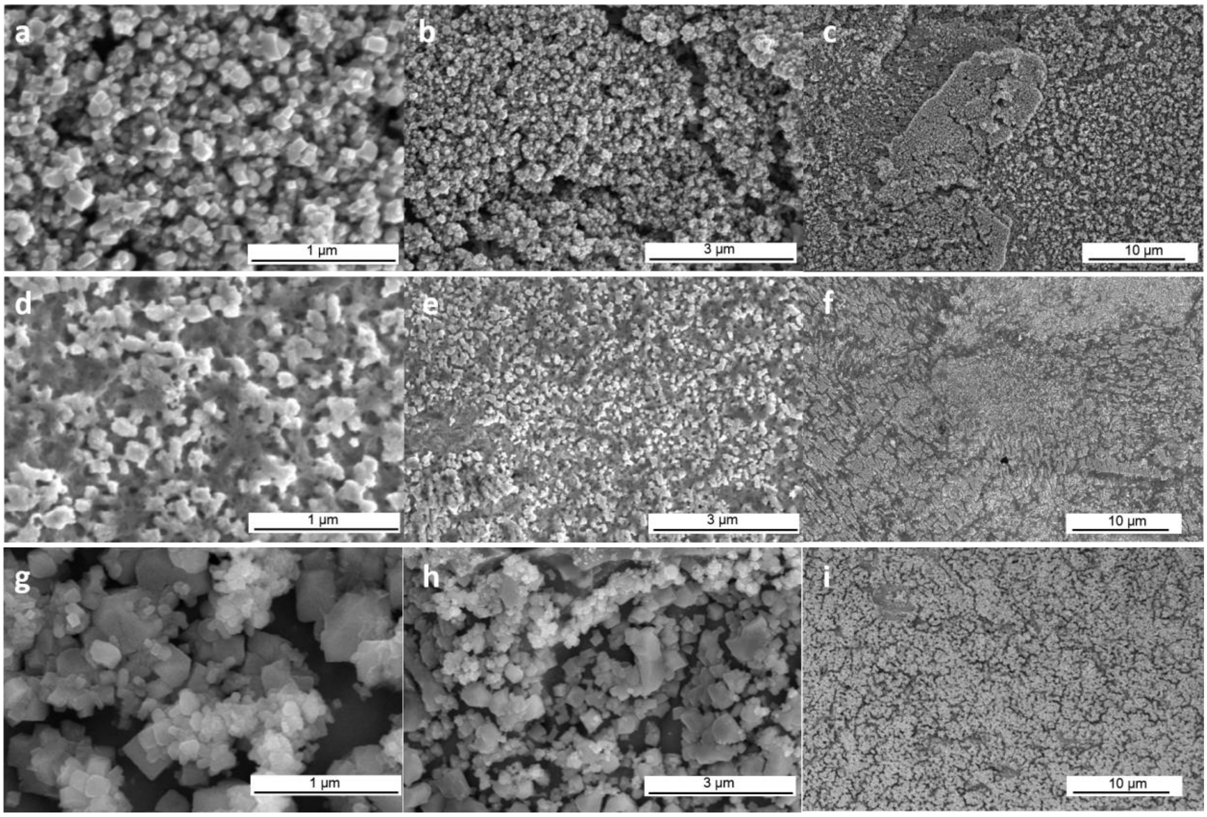

Supplementary Figure 1. SEM images of the electrochemically-treated Cu foil electrode before (a, b, c) and after the CO_2RR in CO_2-saturated 0.1 M $KHCO_3$ electrolyte at –1.0 V_{RHE} (d, e, f) or –0.8 V_{RHE} (g, h, i) for 1 h.

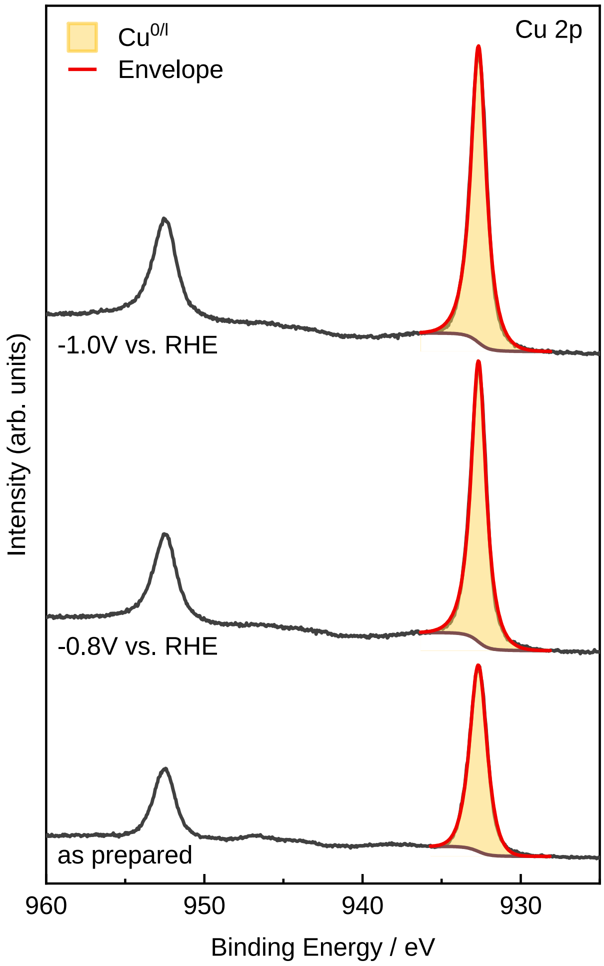

Supplementary Figure 2. Quasi in-situ XPS spectra of the Cu 2p binding energy region of the as-prepared electrochemically-treated Cu foil electrode and the same electrochemically-treated Cu foil electrode after the CO_2RR at –0.8 V_{RHE} and –1.0 V_{RHE} for 1 h in CO_2-saturated 0.1 M $KHCO_3$ electrolyte without air exposure with the corresponding fits for the Cu $2p_{3/2}$ orbital (red line).

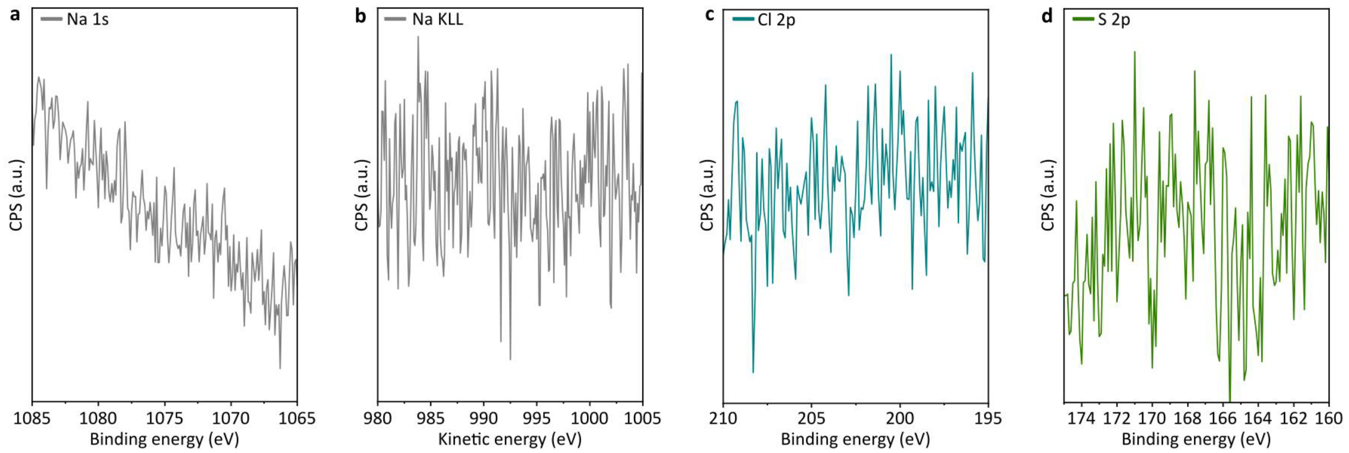

Supplementary Figure 3. The cleanliness of the roughened Cu foil surface was assessed by evaluating the Cl 2p, S 2p as well as Na 1s XPS and Na KLL Auger regions, which represent all elements used during the electropolishing and roughening of the Cu foil. None of the above-mentioned elements could be detected on the surface of the foil after rinsing the sample with ultra-pure water, as done before the CO_2RR measurements.

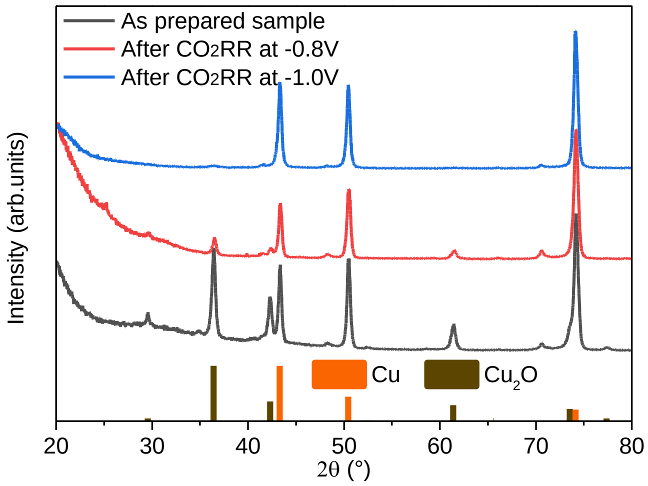

Supplementary Figure 4. XRD pattern of the as-prepared Cu foil electrode before and after CO_2RR in CO_2-saturated 0.1 M $KHCO_3$ electrolyte for 1 h. The intensity of the Cu(200) reflection slightly increased compared to the (111) reflection. However, the absence of a clearly reflected X-ray beam under total reflection condition suggests an information depth above 100 nm and thus, we cannot assign any variations in the XRD pattern to morphological differences in the near-surface region of the electrodes.

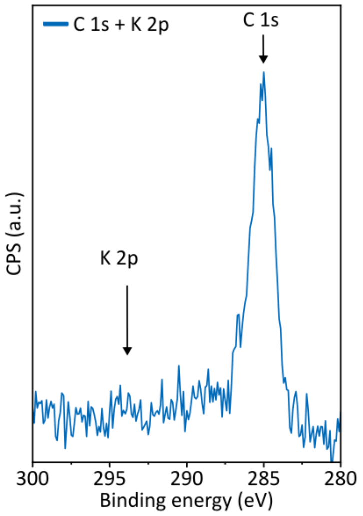

Supplementary Figure 5. XPS regions of K 2p and C 1s were acquired after a quasi-in situ CO_2RR experiment, which revealed that no potassium residues could be detected from the surface after rinsing the sample in ultra-pure water.

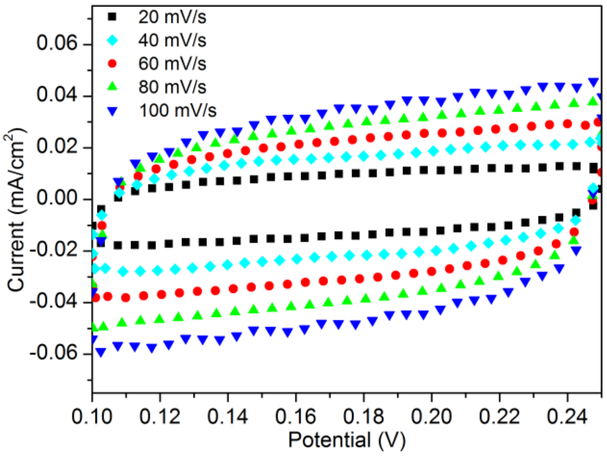

Supplementary Figure 6. Cyclic voltammetry of the electrochemically-treated Cu foil electrode from 0.10 to 0.25 V_{RHE} at scan rates of 20, 40, 60, 80, and 100 mV/s in a CO_2-saturated 0.1 M $KHCO_3$ solution after 1 h of CO_2RR test. The capacitance value of electrochemically-treated Cu foil is 0.395 mF/cm^2, and the electropolished Cu foil is 0.027 mF/cm^2.

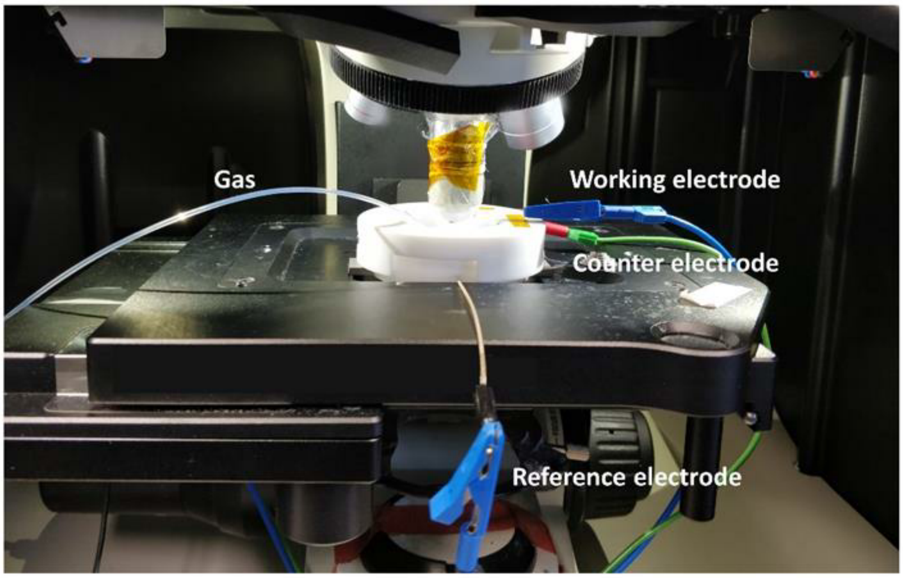

Supplementary Figure 7. The photograph of the in-situ Raman set-up.[9]

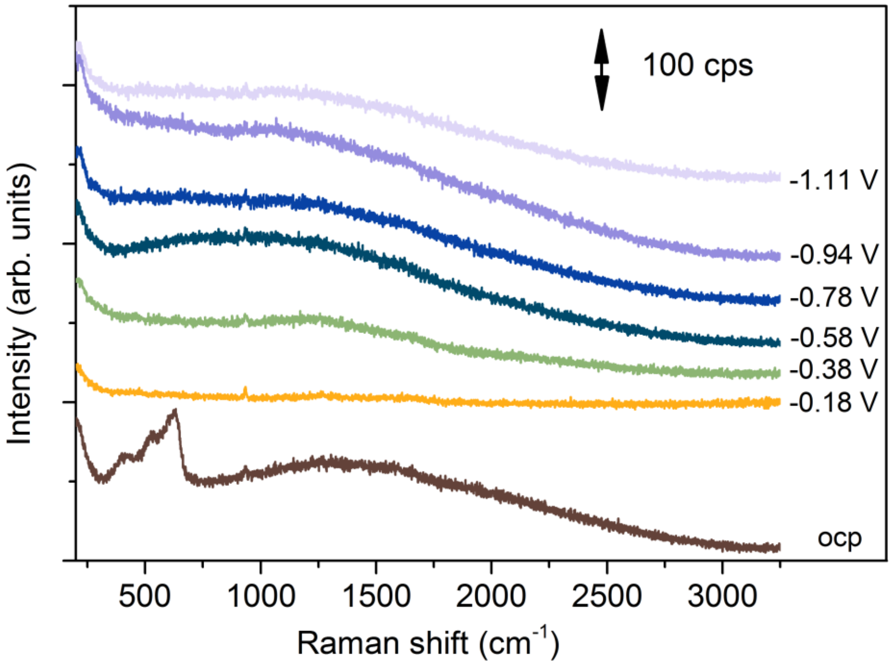

Supplementary Figure 8. Electrochemical Raman spectra of the electrochemically-polished Cu foil electrodes at potentials from OCP to –1.11 V_{RHE} in an Ar-saturated 0.1 M $NaClO_4$ solution.

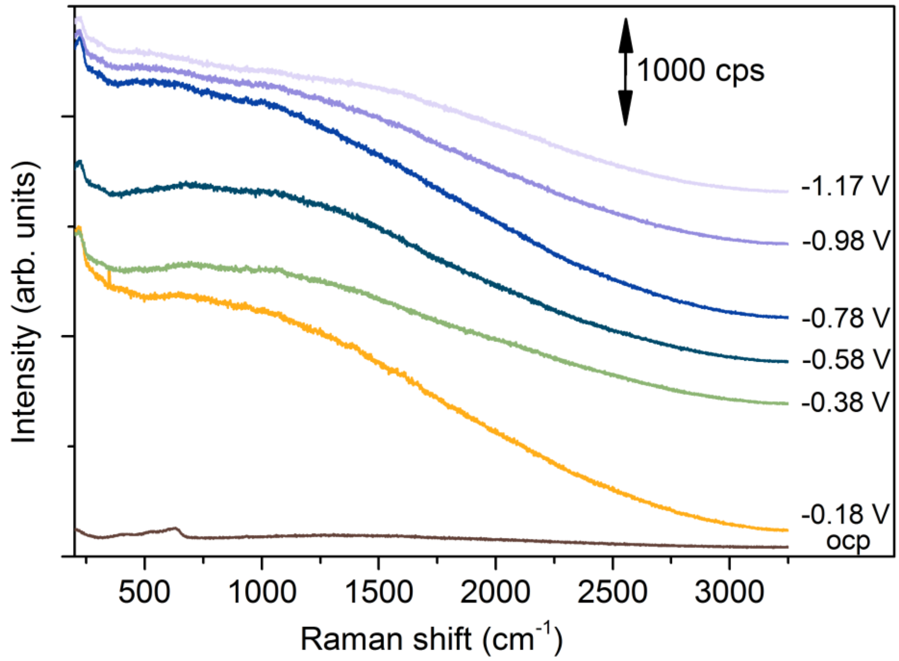

Supplementary Figure 9. Electrochemical surface-enhanced Raman spectra of the electrochemically-treated Cu foil electrode at potentials from OCP to –1.17 V_{RHE} in an Ar-saturated 0.1 M $NaClO_4$ solution.

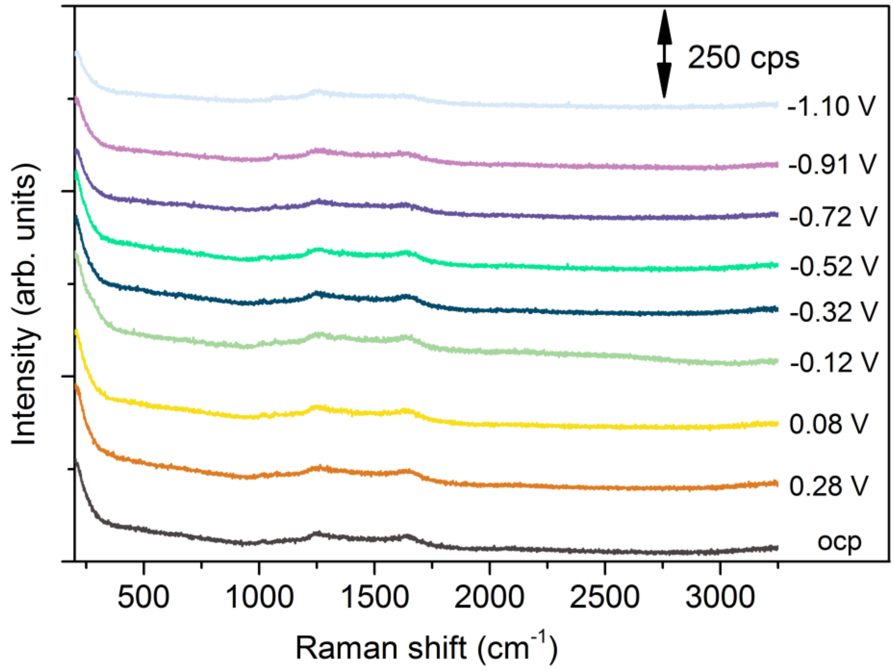

Supplementary Figure 10. Electrochemical Raman spectra of the electrochemically-polished Cu foil electrode at potentials from OCP to –1.10 V_{RHE} in an Ar-saturated 0.1 M $KHCO_3$ solution.

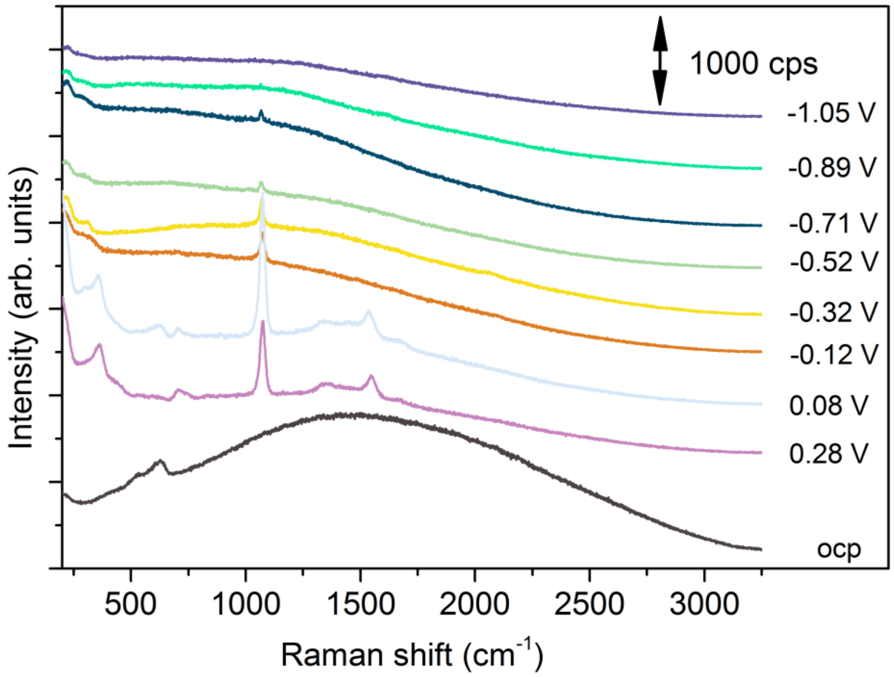

Supplementary Figure 11. Electrochemical surface-enhanced Raman spectra of the electrochemically-treated Cu foil electrode at potentials from OCP to –1.05 V_{RHE} in an Ar-saturated 0.1 M $KHCO_3$ solution.

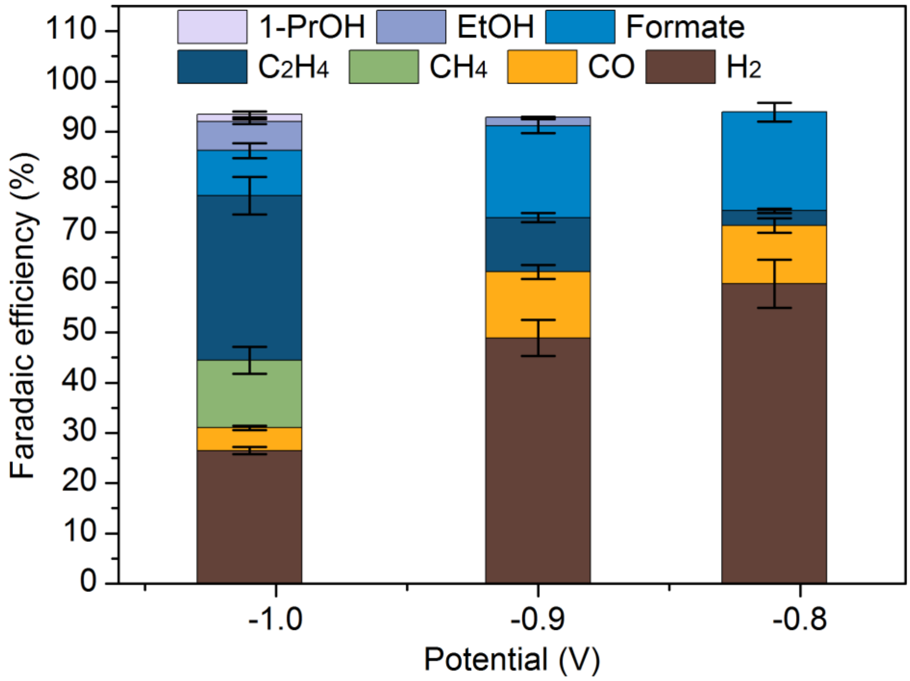

Supplementary Figure 12. Potential-dependent Faradaic efficiency of the electrochemically-treated Cu foil electrode in CO_2-saturated 0.1M $NaClO_4$. The error bars correspond to the standard deviation (SD) of three independent measurements. Data are given as averages ± SD.

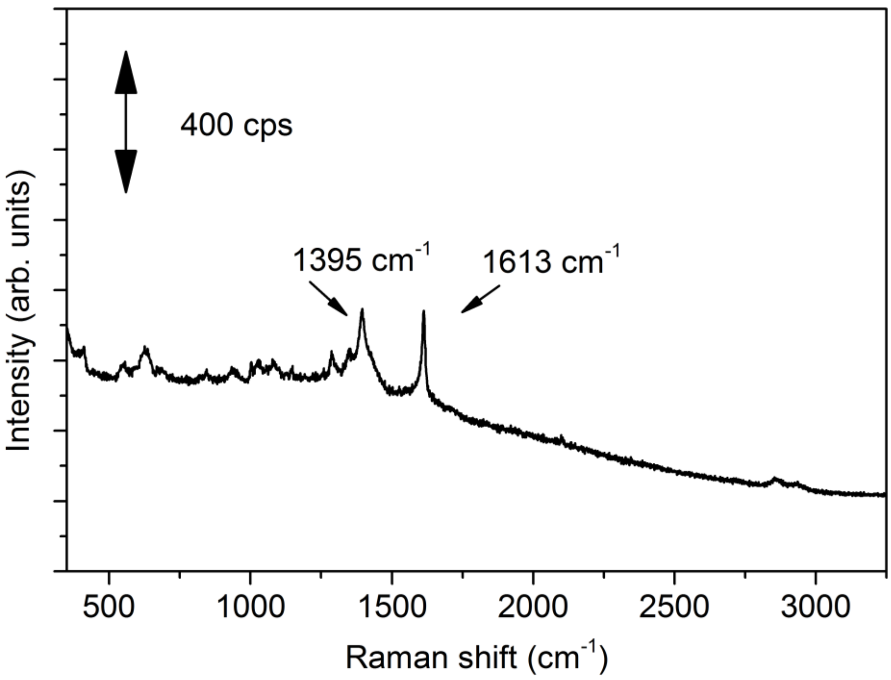

Supplementary Figure 13. Electrochemical surface-enhanced Raman spectrum of an electrochemically-treated Cu foil electrode at –0.4 V_{RHE} in an Ar-saturated 0.1 M 0.1 M $NaClO_4$ with 1 mM HCOOH solution.

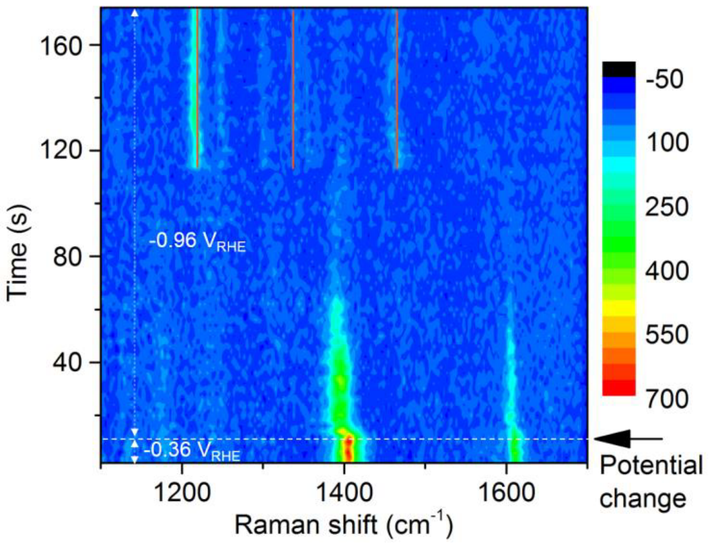

Supplementary Figure 14. Time-dependent Raman spectra of an electrochemically-treated Cu foil acquired during the potential changing from –0.36 V_{RHE} to –0.96 V_{RHE} in CO_2-saturated 0.1 M $NaClO_4$ electrolyte. The acquisition time was 2 s. Time-dependent Raman spectroscopy measurements were performed to track the initial changes under CO_2RR with a time resolution of 2 s. Although the data quality was low for the time-resolved experiments, it can be observed that the peaks of the *$HCOO^-$/*HCOOH at ~1390 cm^{-1} and 1410 cm^{-1} as well as of the C-C coupling intermediate $OCHCH_2$ at about 1200 cm^{-1}, 1320 cm^{-1} and 1450 cm^{-1} appear simultaneously. Interestingly, when we changed the potential from –0.36 V_{RHE} to –0.96 V_{RHE}, the peaks assigned to *$HCOO^-$/*HCOOH immediately shifted to lower wavenumbers due to the electrochemical Stark effect and gradually decreased in intensity. It takes 80 s after applying the CO_2RR potential until these peaks disappeared completely and the peaks assigned to the C-C coupling intermediate appeared.

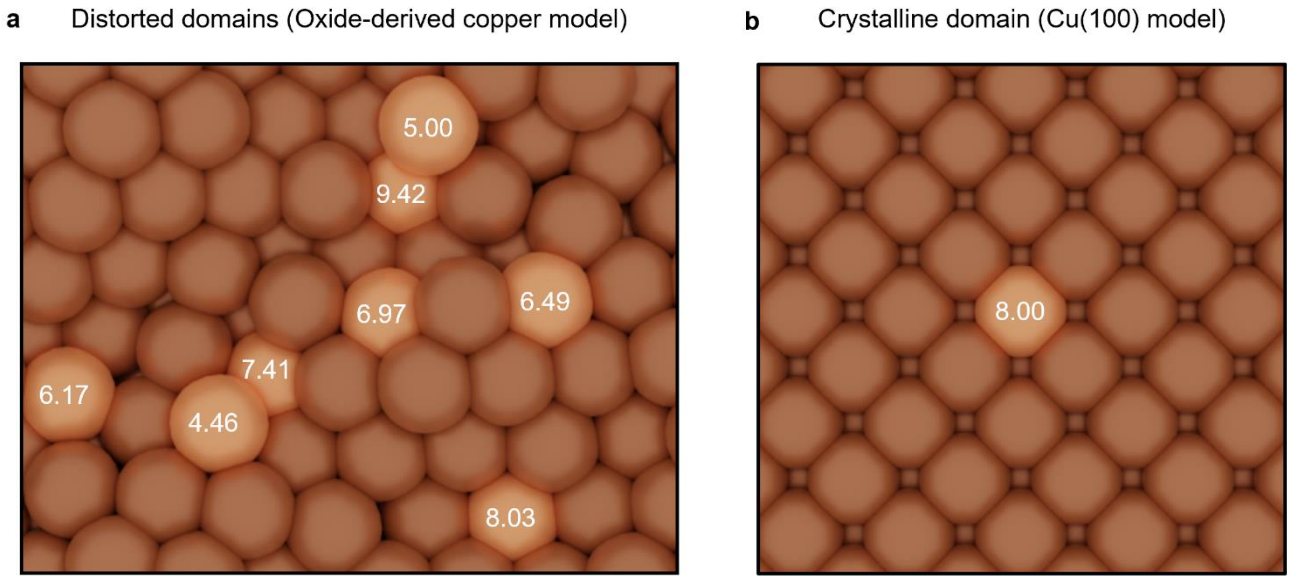

Supplementary Figure 15. Models for (a) distorted domains (Ref.[10]) and (b) crystalline domain, i.e. Cu(100) p(3×3), with active sites highlighted in light brown and Cu-Cu coordination numbers indicated in white.

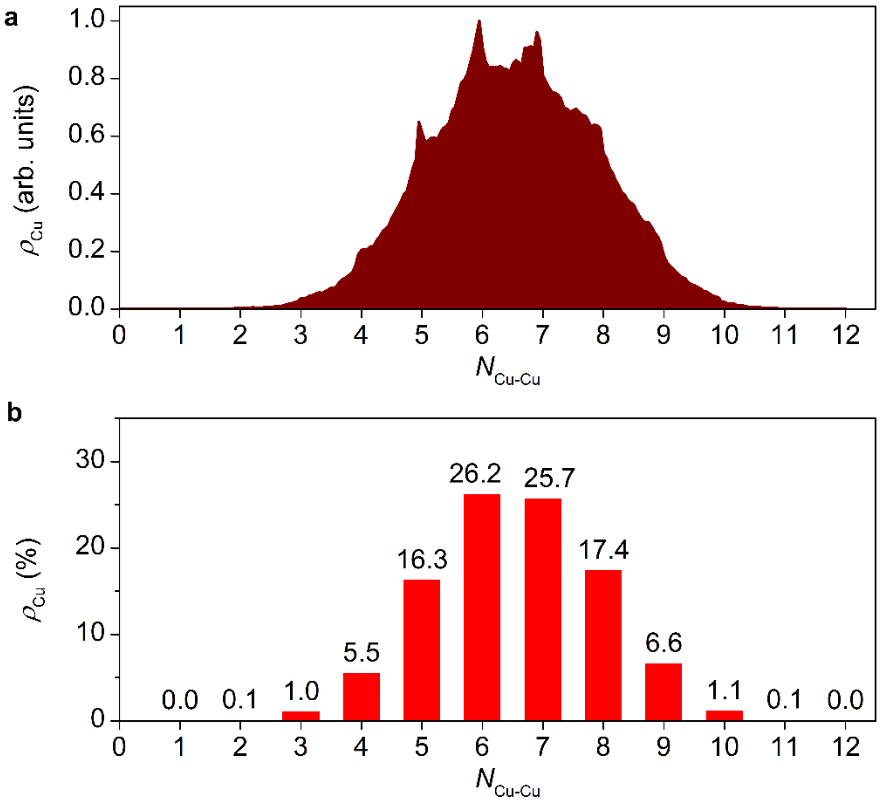

Supplementary Figure 16. (a) Cu-Cu coordination number cumulative map for surface sites of the OD-Cu model during 5 ps ab initio molecular dynamics simulation.[8] (b) Abundance (%) of Cu sites with given coordination numbers, calculated as integral of (a) within (–0.5 N_{Cu-Cu}, +0.5 N_{Cu-Cu}), e.g. 3.5, 4.5 for N_{Cu-Cu} = 4.

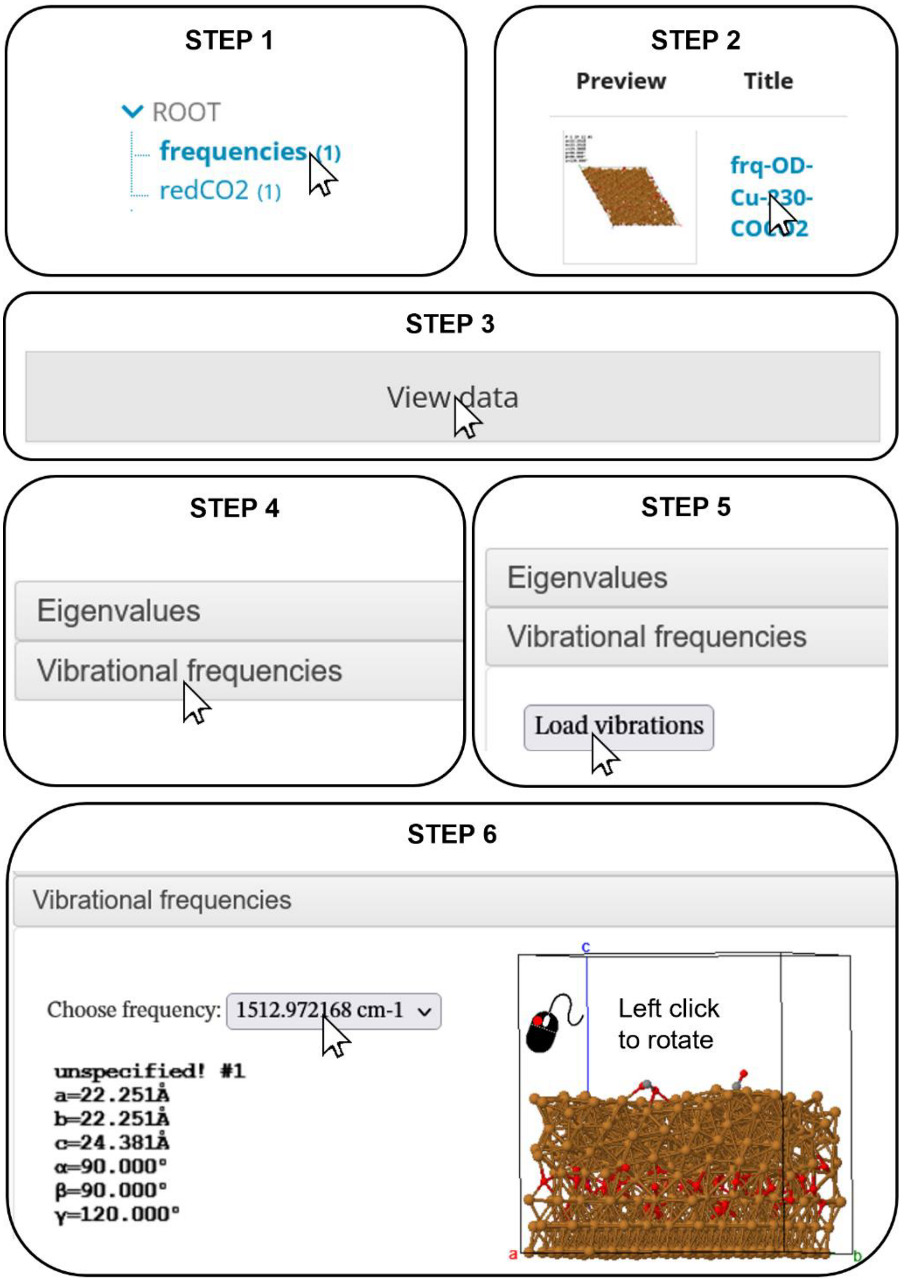

Supplementary Figure 17. Tutorial on how to visualize vibrational frequencies for a given reaction intermediate on ioChem-BD.[11]

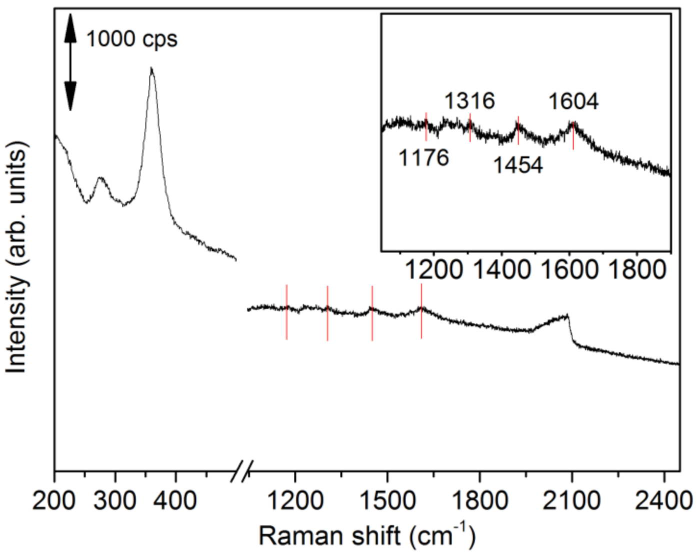

Supplementary Figure 18. In-situ Raman spectra of an electrochemically-treated Cu foil acquired during CO electrochemical reduction at about –1.0 V_{RHE} in a CO-saturated 0.1 M $NaClO_4$ electrolyte.

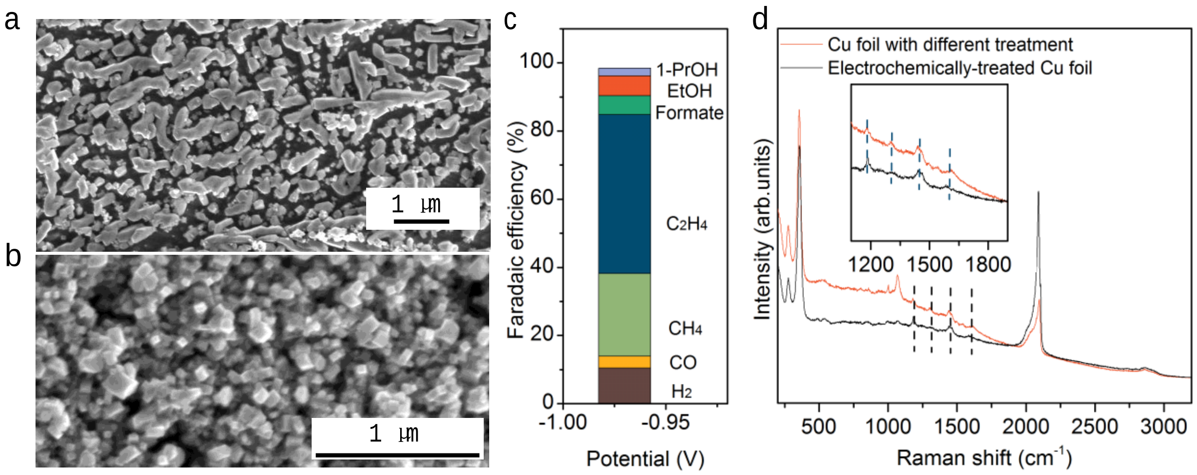

Supplementary Figure 19. (a) SEM image of a Cu foil electrode with that was subjected to a different electrochemical pre-treatment as that utilized in the rest of the manuscript (2 cyclic voltammograms, gas-saturated 0.1 M $NaClO_4$, 10 mV/s, −1.3 V_{RHE} to +0.5 V_{RHE}), (b) SEM image of Cu foil electrode used in the main text as comparison. (c) Faradaic efficiency of the differently pre-treated Cu foil electrode in CO_2-saturated 0.1M $KHCO_3$ electrolyte for 1 h at –0.97 V_{RHE}. (d) Raman spectra of the two differently pre-treated Cu foils acquired at –0.95 V_{RHE} during CO_2RR in a CO_2-saturated 0.1 M $NaClO_4$ electrolyte.

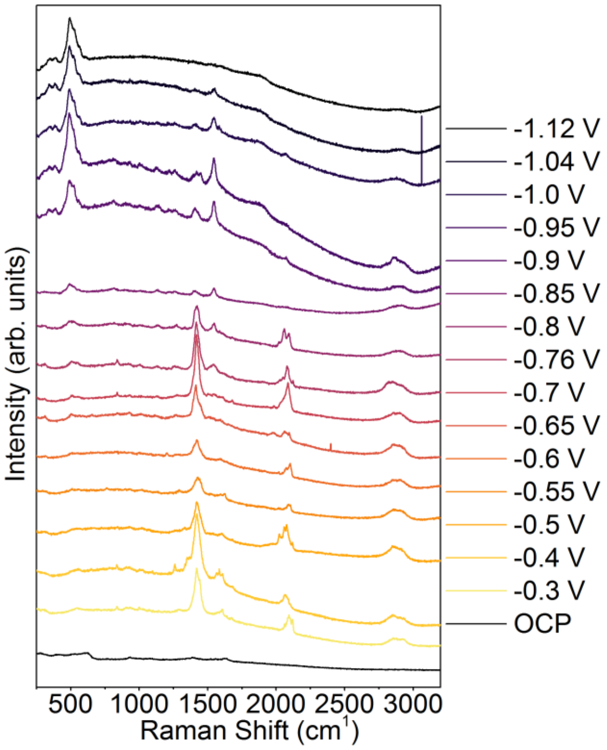

Supplementary Figure 20. Surface-enhanced Raman spectra of an electrochemically-treated Cu foil electrode acquired during glyoxal reduction at potentials from OCP to –1.1 V_{RHE} in an Ar-saturated 0.1 M $NaClO_4$ solution with 0.05 M glyoxal.

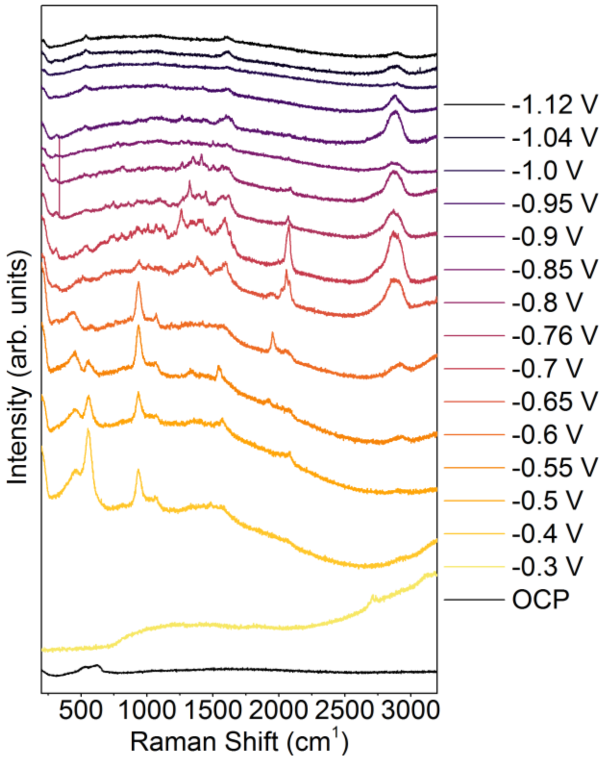

Supplementary Figure 21. Surface-enhanced Raman spectra of an electrochemically-treated Cu foil electrode acquired during ethanol reduction at potentials from OCP to –1.1 V_{RHE} in an Ar-saturated 0.1 M $NaClO_4$ solution with 0.05 M ethanol.

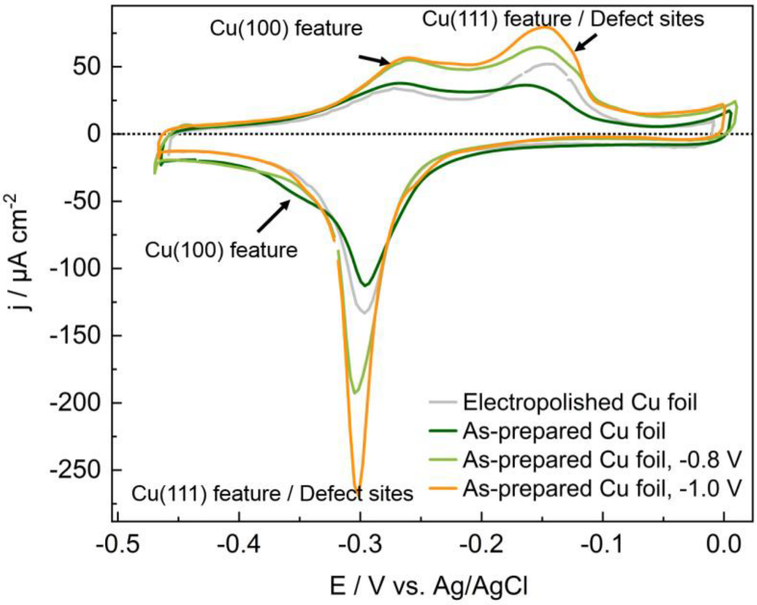

Supplementary Figure 22. The Pb UPD experiments of the Cu foil before and upon CO_2RR at –0.8 and –1.0 V_{RHE} for one hour. Pb UPD was conducted in a Ar-saturated solution of 0.1 M $NaClO_4$, 10 mM $HClO_4$, and 3 mM $Pb(ClO_4)_2$. The scan rate is 10 mV/s. From the Pb UPD experiments of the Cu surfaces before and upon 1h CO_2RR at –0.8 and –1.0 V, it is clear that the anodic feature at about –0.16 $V_{Ag/AgCl}$ (which can be correlated to defective Cu sites or Cu(111))[12,13] is suppressed as the cathodic feature at –0.35 $V_{Ag/AgCl}$ (which can be correlated to Cu(100) sites)[12,13] becomes more pronounced after electrochemically-treating the Cu Foil in 0.1M $NaClO_4$. However, these features recover and become more obvious after CO_2RR at –0.8 and –1.0 V. Additionally, compared with the –0.8 V case, there are more defective Cu sites on the electrochemically-treated Cu electrode after CO_2RR at –1.0 V, in agreement to the cyclic voltammetry experiments.

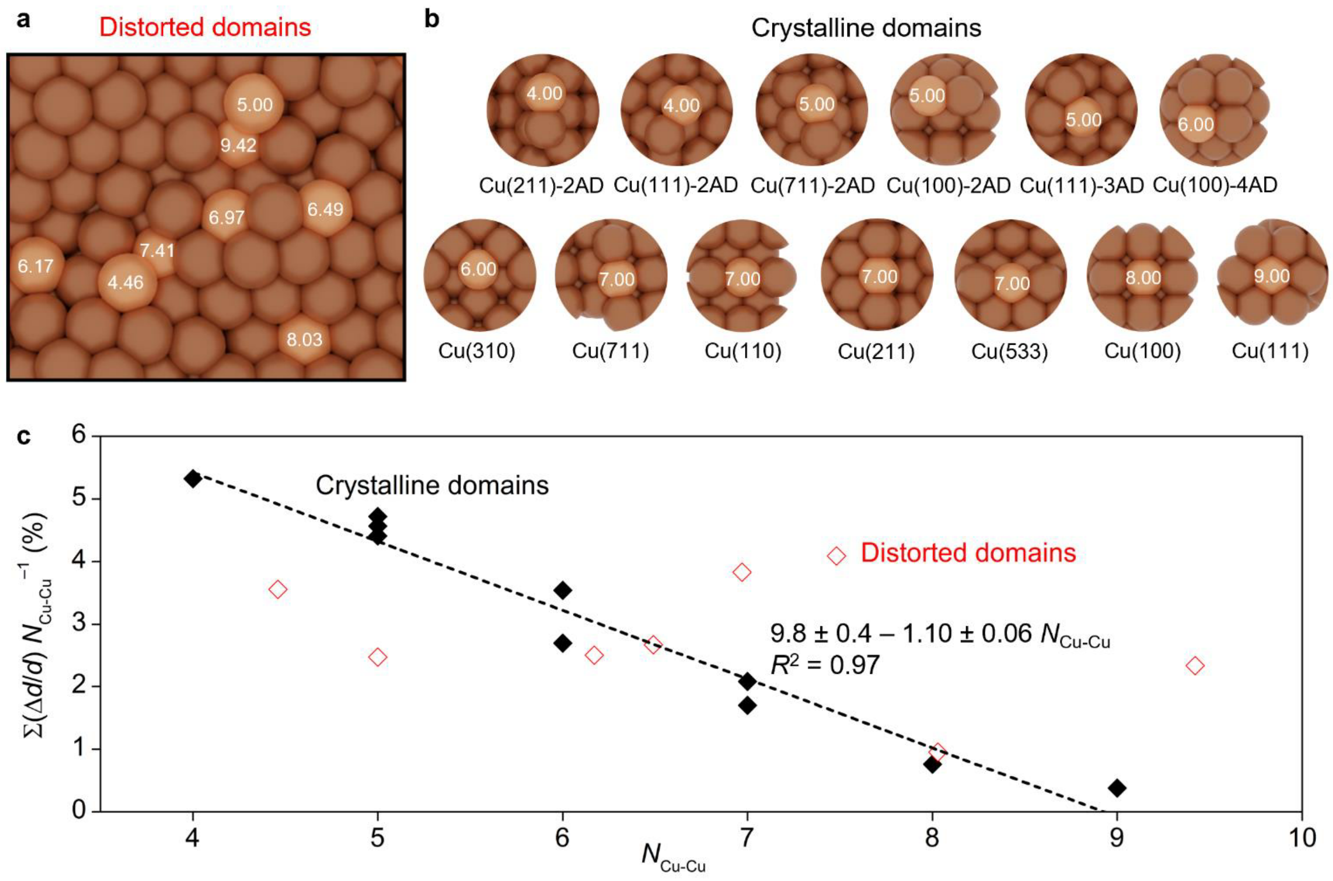

Supplementary Figure 23. (a) Models for distorted domains (Ref.[10]) and (b) crystalline facet, i.e. Cu(100), with active sites highlighted in light brown and Cu-Cu coordination numbers indicated in white. (c) Correlation between $\Sigma(\Delta d/d)\ N_{Cu\text{-}Cu}^{-1}$ and $N_{Cu\text{-}Cu}$ for crystalline (black filed points) and distorted sites (red empty points).

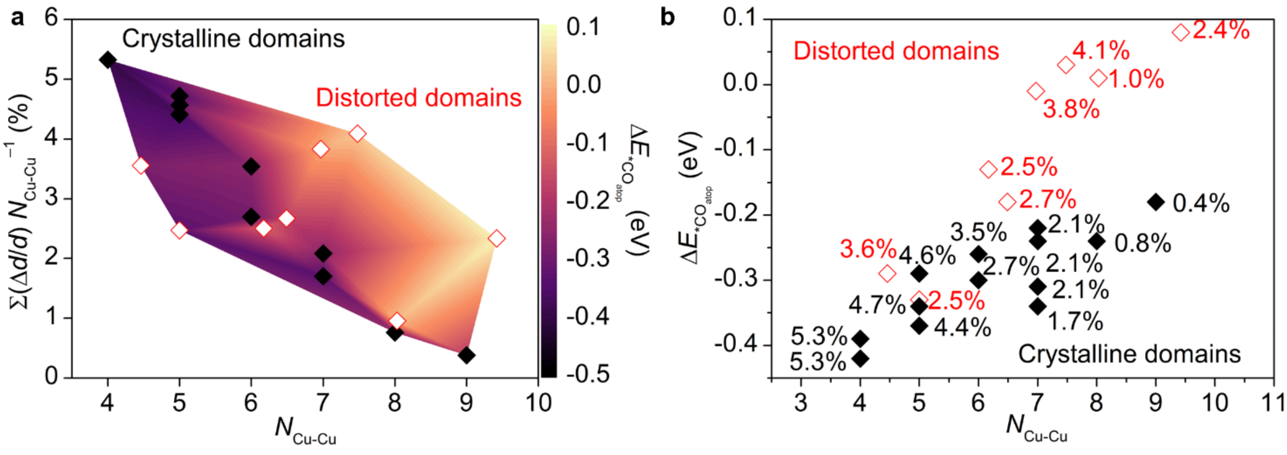

Supplementary Figure 24. (a) DFT adsorption energy for *CO atop on crystalline (black filled points, Supplementary Figure 15b) and distorted domains (red empty points, Supplementary Figure 15a) vs Cu-Cu coordination number ($N_{Cu\text{-}Cu}$, x-axis) and compressive strain normalized by Cu coordination number ($\Sigma(\Delta d/d)\ N_{Cu\text{-}Cu}^{-1}$, y-axis). Darker areas correspond to stronger binding energy. (b) Correlation of *CO atop binding energies on crystalline and distorted sites vs Cu-Cu coordination number. Values of compressive strain normalized by Cu coordination number are included as labels.

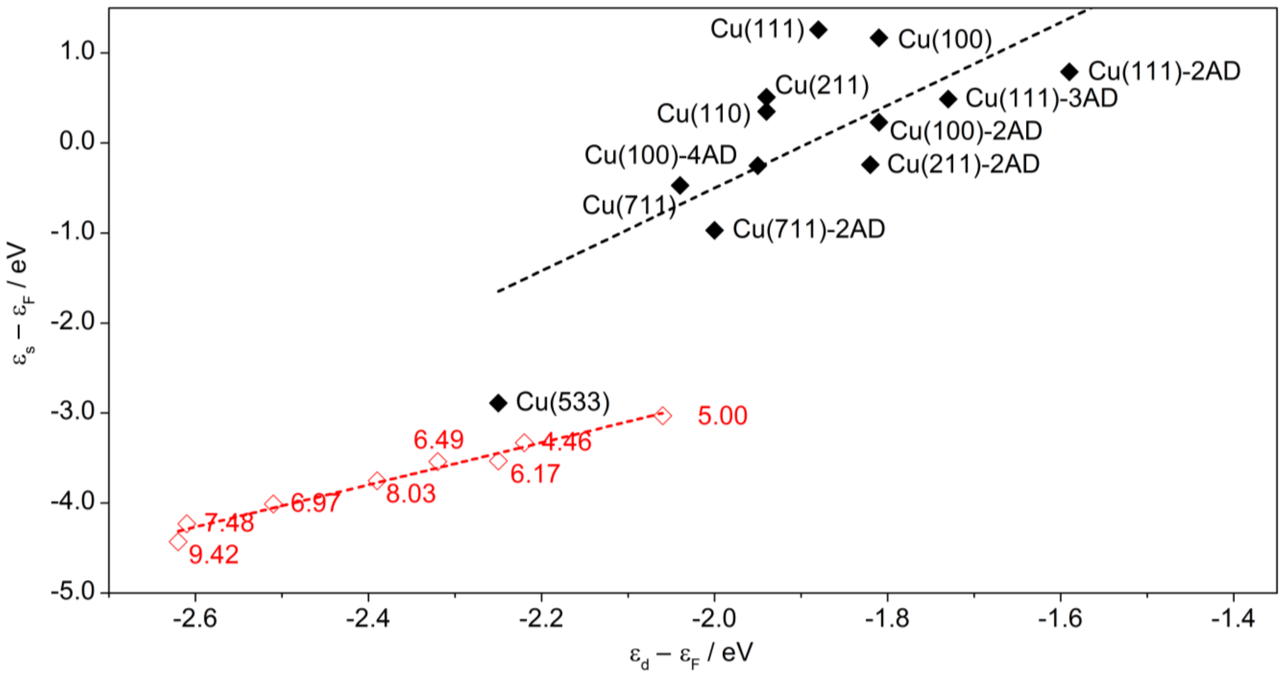

Supplementary Figure 25. Linear correlation between s-band center (vs Fermi energy, $\varepsilon_s - \varepsilon_F$) and d-band center (vs Fermi energy, $\varepsilon_d - \varepsilon_F$) for distorted sites (red empty data points) and crystalline domains (black filled data points). Fitting parameters are reported in Supplementary Table 17.

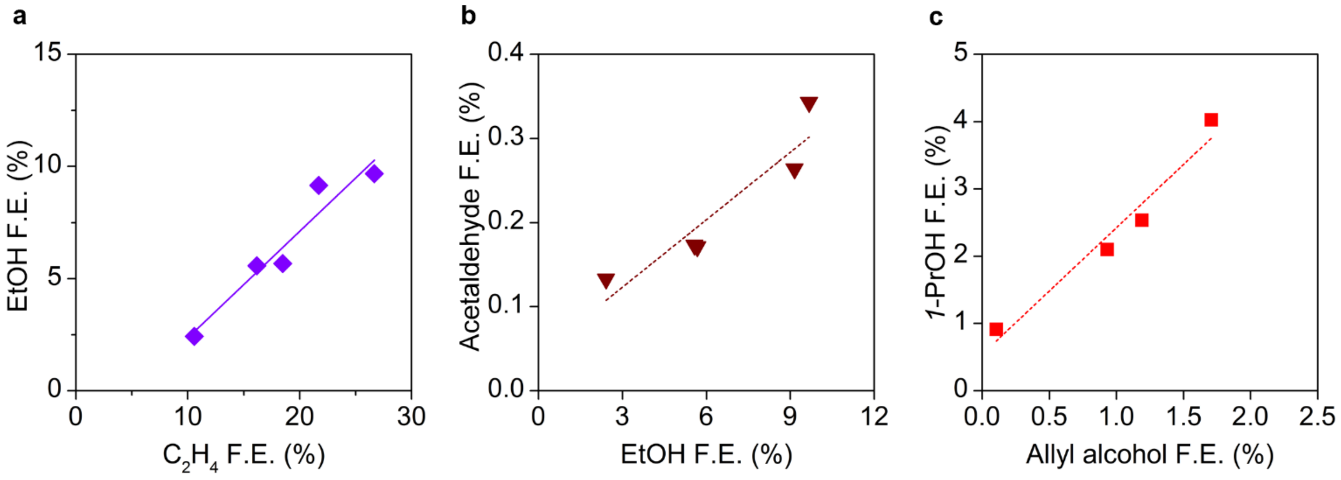

Supplementary Figure 26. (a) Correlation between ethanol and ethylene selectivities. (b) Correlation between acetaldehyde and ethanol selectivities. (c) Correlation between 1-propanol and allyl alcohol selectivities. Values of Faradaic efficiencies were measured between –0.75 V vs RHE and –1.2 V vs RHE during CO_2 electrolysis on a 1.5 cm × 3 cm Cu working electrode in 0.1 M solution of $KHCO_3$ (bulk pH = 6.8).[4] Fitting parameters are reported in Supplementary Table 19.

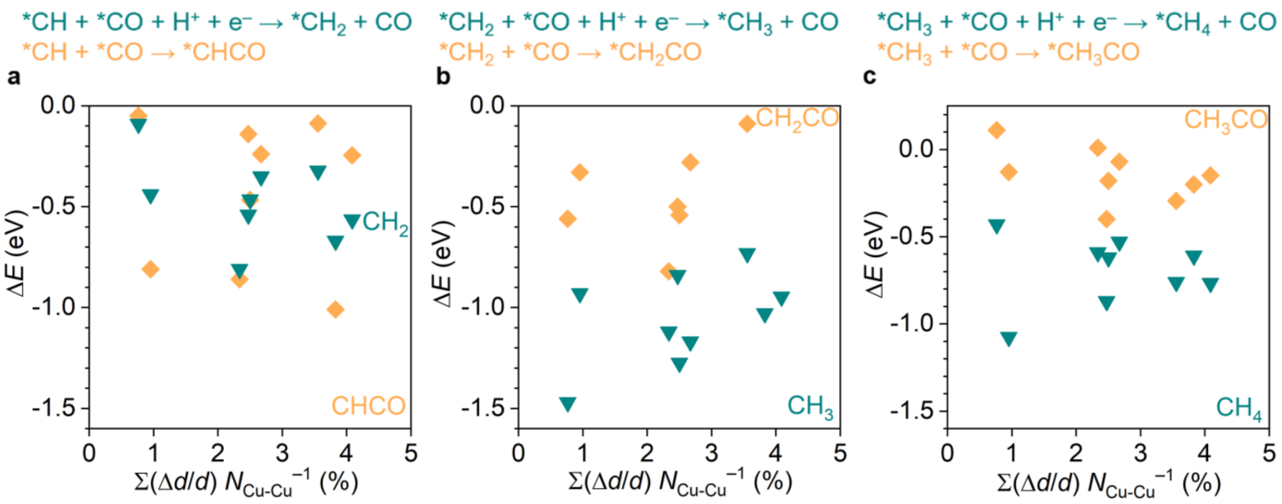

Supplementary Figure 27. DFT energy associated with the CH_x-CO coupling step toward ethanol precursors (cyan) vs CH_x protonation to methane on distorted domains (orange). In the figure x = 1, 2, 3 for panels a, b, c, respectively.

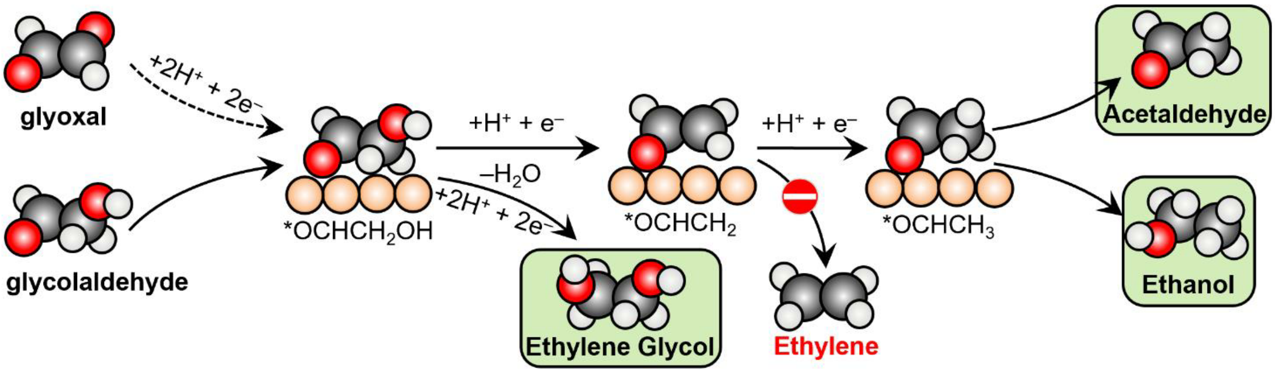

Supplementary Figure 28. Reaction scheme for glyoxal and glycolaldehyde reduction toward acetaldehyde and ethanol, as proposed in Ref. [5].

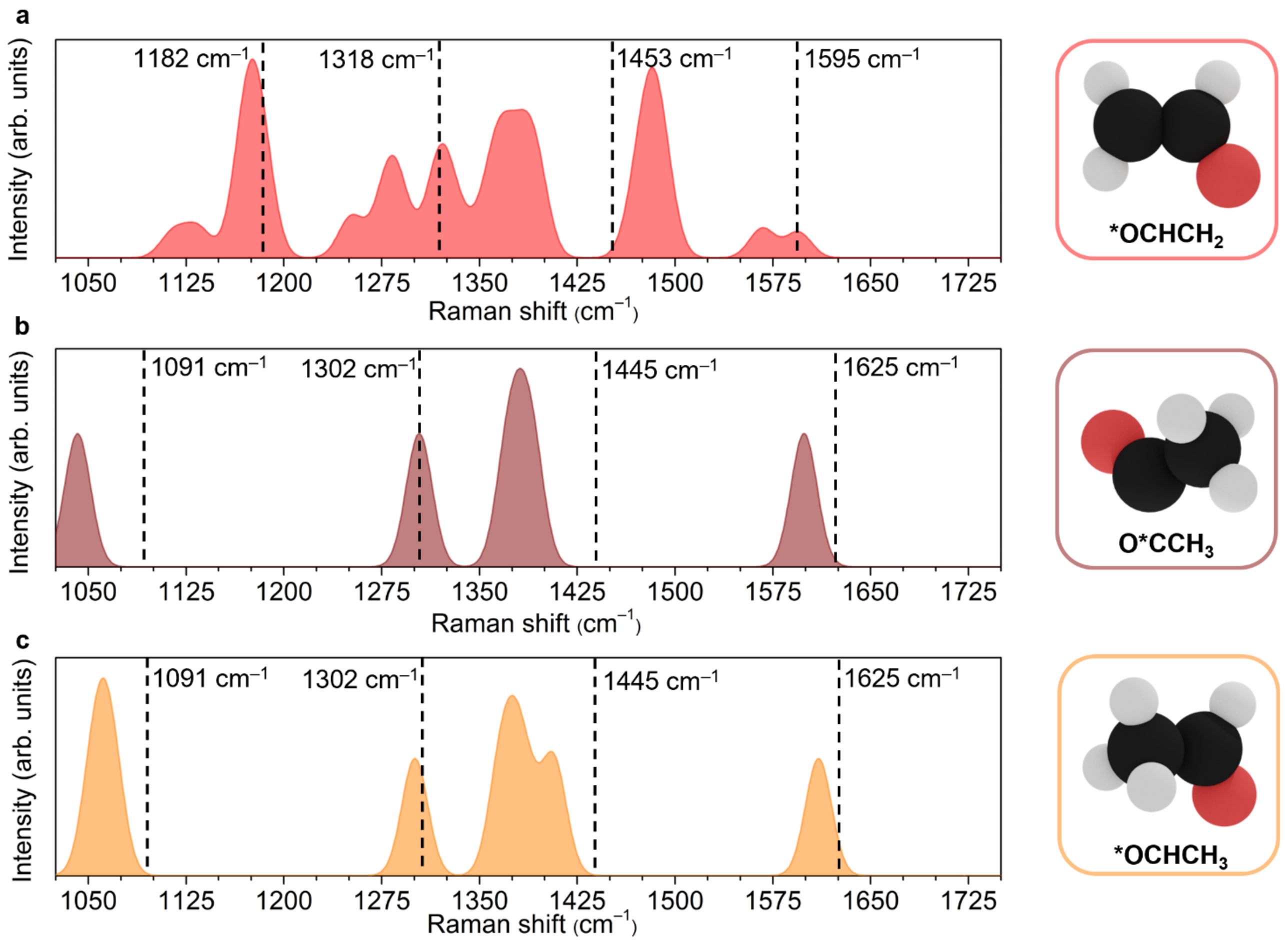

Supplementary Figure 29. Vibrational frequencies for intermediates toward ethanol and 1-propanol: (a) $*OCHCH_2$ on distorted Cu sites proposed in this study, and (b) $O*CCH_3$ on Cu(100), proposed in Refs.[14,15]. (c) Vibrational frequencies of adsorbed acetaldehyde on Cu(100) are reported for comparison. Experimental signals are highlighted by vertical dashed lines and are taken from this study (Fig.2c), Ref.[[15]] (1091 cm^{-1}), and Ref.[14]] (1302 cm^{-1}, 1445 cm^{-1}, 1625 cm^{-1}). Theoretical Raman spectra were achieved by applying a smearing of 10 cm^{-1} on each DFT frequency and overlapping the resulting peaks. Vibrational modes can be visualized at links (a) https://iochem-bd.iciq.es/browse/handle/100/40738, (b) https://iochem-bd.iciq.es/browse/handle/100/59928, and (c) https://iochem-bd.iciq.es/browse/handle/100/40732 respectively.

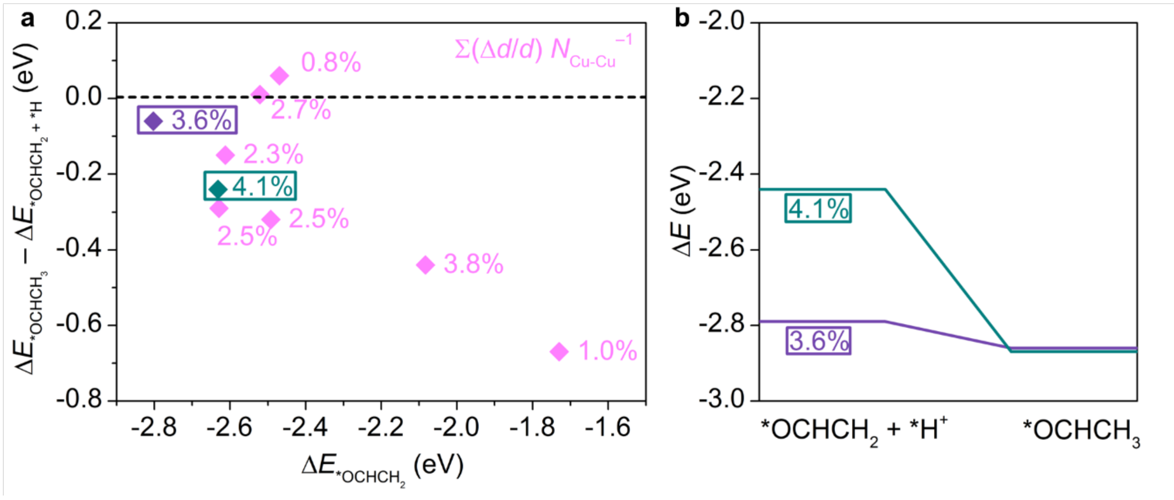

Supplementary Figure 30. (a) DFT energy associated with $*OCHCH_2$ protonation to $*OCHCH_3$ on distorted domains. Data labels indicate the associated 3-D strain of each site. The 3.6% and 4.1% 3-D strain data points are highlighted by purple and green boxes respectively. (b) Gibbs free energy diagrams for $*OCHCH_2$ protonation to $*OCHCH_3$ on previously selected highly strained active sites.

Supplementary Tables

Supplementary Table 1: PBE-D2 vibrational frequencies (in cm^{-1}) calculated for $*CO_3^{2-}$ on different actives sites (Supplementary Figure 15). Gray area indicates unstable configurations. Vibrational modes can be visualized in the ioChem-BD database (see Supplementary Figure 17 for details).[16] For instance, vibrational modes of adsorbates on Cu(100) are accessible at Ref. [17].

Model	Oxide-derived copper								Cu(100)
$N_{Cu\text{-}Cu}$	4.46	5.00	6.17	6.49	6.97	7.48	8.03	9.42	8.00
CO_3^{2-}	1637	1621	1676			1705		1524	1712
	1079	1124	993			1166		1214	979
	845	827	908			763		939	906
	769	764	769			751		772	775
	674	670	652			667		687	654
	592	562	599			587		633	601
	316	318	271			304		282	284
	288	238	253			294		255	279
	220	155	226			184		215	246
	103	127	202			167		181	211
	71	79	113			104		167	101
	53	48	89			71		109	85

Supplementary Table 2: PBE-D2 vibrational frequencies (in cm^{-1}) calculated for $HCOO^-$ on different actives sites (Supplementary Figure 15). Vibrational modes can be visualized in the ioChem-BD database (see Supplementary Figure 17 for details).[16] For instance, vibrational modes of adsorbates on Cu(100) are accessible at Ref. [18].

Model	Oxide-derived copper								Cu(100)
$N_{Cu\text{-}Cu}$	4.46	5.00	6.17	6.49	6.97	7.48	8.03	9.42	8.00
$HCOO^-$	2951	2936		2948	2953	2939	2945	2932	2913
	1540	1543		1538	1517	1540	1526	1541	1664
	1341	1350		1327	1318	1340	1321	1350	1300
	1317	1318		1316	1300	1318	1308	1317	1112
	985	990		995	985	989	985	992	981
	720	712		745	739	728	740	713	708
	341	335		331	303	339	307	336	217
	294	304		317	291	316	296	308	178
	279	276		304	263	288	265	278	112
	125	124		125	119	124	111	122	79
	115	110		108	90	109	105	120	40
	91	103		93	77	88	92	99	–

Supplementary Table 3: PBE-D2 vibrational frequencies (in cm^{-1}) calculated for HCOOH on different actives sites (Supplementary Figure 15). Vibrational modes can be visualized in the ioChem-BD database (see Supplementary Figure 17 for details).[16] For instance, vibrational modes of adsorbates on Cu(100) are accessible at Ref. [19].

Model	Oxide-derived copper								Cu(100)
$N_{Cu\text{-}Cu}$	4.46	5.00	6.17	6.49	6.97	7.48	8.03	9.42	8.00
HCOOH	3101	3094	3039	3029	3046	3530	3147	3078	3253
	3024	3021	2993	2963	3001	2997	3010	3019	2972
	1650	1645	1646	1649	1644	1768	1650	1642	1700
	1356	1360	1354	1353	1359	1344	1360	1361	1352
	1303	1301	1300	1292	1299	1221	1289	1304	1266
	1144	1140	1144	1145	1146	1006	1135	1139	1110
	994	999	995	996	996	975	993	996	999
	703	717	708	714	720	663	680	714	697
	655	651	652	650	655	601	644	650	628
	246	258	238	228	248	195	218	264	140
	227	236	220	207	232	146	204	229	104
	130	118	135	143	144	85	140	143	73
	97	103	95	98	95	56	105	98	49
	74	82	92	69	81	38	81	88	39
	44	75	70	28	55		66	63	14

Supplementary Table 4: PBE-D2 vibrational frequencies (in cm^{-1}) calculated for *OCCO on different actives sites (Supplementary Figure 15). Gray area indicates unstable configurations. Vibrational modes can be visualized in the ioChem-BD database (see Supplementary Figure 17 for details).[16] For instance, vibrational modes of adsorbates on Cu(100) are accessible at Ref. [20].

Model	Oxide-derived copper								Cu(100)
$N_{Cu\text{-}Cu}$	4.46	5.00	6.17	6.49	6.97	7.48	8.03	9.42	8.00
*OCCO		1996		1773	1689	1700		1481	2015
		1399		1382	1456	1412		1434	1210
		882		690	595	683		766	861
		541		568	472	587		549	606
		362		437	320	511		482	397
		319		331	285	342		337	367
		306		299	252	304		304	327
		285		229	202	239		271	296
		145		171	189	198		187	258
		95		136	104	144		126	152
		80		83	81	129		113	82
		45		25	49	74		100	52

Supplementary Table 5: PBE-D2 vibrational frequencies (in cm^{-1}) calculated for *OCCOH on different actives sites (Supplementary Figure 15). Vibrational modes can be visualized in the ioChem-BD database (see Supplementary Figure 17 for details).[14] For instance, vibrational modes of adsorbates on Cu(100) are accessible at Ref. [21].

Model	Oxide-derived copper								Cu(100)
$N_{Cu\text{-}Cu}$	4.46	5.00	6.17	6.49	6.97	7.48	8.03	9.42	8.00
*OCCOH	3493	3691	3642	3559	3464	3590	3519	3532	3626
	1577	1965	2027	1531	1528	1373	1317	1952	2022
	1232	1321	1217	1255	1251	1262	1256	1293	1189
	1054	1247	1163	1056	1096	1082	1094	1230	1179
	843	884	814	929	868	891	946	850	844
	726	552	599	697	726	711	760	553	599
	542	363	494	477	570	464	554	404	486
	476	336	351	439	456	407	461	392	314
	297	255	300	318	319	299	318	371	293
	265	237	282	273	277	294	302	245	284
	239	196	249	224	235	267	264	220	215
	199	177	145	183	220	199	225	197	136
	148	109	134	132	143	165	164	134	115
	129	69	87	101	113	132	140	101	80
	81	57	64	78	93	106	118	94	65

Supplementary Table 6: PBE-D2 vibrational frequencies (in cm^{-1}) calculated for $*OCCO_2$ on different actives sites (Supplementary Figure 15). Gray area indicates unstable configurations. Vibrational modes can be visualized in the ioChem-BD database (see Supplementary Figure 17 for details).[16] For instance, vibrational modes of adsorbates on Cu(100) are accessible at Ref. [22].

Model	Oxide-derived copper								Cu(100)
$N_{Cu\text{-}Cu}$	4.46	5.00	6.17	6.49	6.97	7.48	8.03	9.42	8.00
$*OCCO_2$			1704	1759	1704	1733	1768		1707
			1636	1747	1680	1513	1750		1684
			1158	1160	1153	1203	1128		1078
			789	673	753	772	702		781
			734	655	700	746	641		727
			561	617	581	562	529		562
			530	416	541	541	468		531
			335	305	339	394	312		355
			226	296	251	293	237		248
			225	237	218	235	198		234
			210	182	202	193	188		194
			117	77	114	176	77		148
			92	69	91	121	69		96
			66	68	76	80	52		89
			37	62	67	73	23		58

Supplementary Table 7: PBE-D2 vibrational frequencies (in cm^{-1}) calculated for $*OCHCH_2$ and $*OCHCH_3$ on different actives sites (Supplementary Figure 15). Vibrational modes can be visualized in the ioChem-BD database (see Supplementary Figure 17 for details).[16] Vibrational modes of adsorbates on Cu(100) are accessible at Refs. [23,24].

Model	Oxide-derived copper								Cu(100)
N_{Cu-Cu}	4.46	5.00	6.17	6.49	6.97	7.48	8.03	9.42	8.00
$*OCHCH_2$	3125	3124	3135	3109	3178	3118	3167	3111	3150
	3013	3027	3028	3045	3073	3010	3056	3014	3040
	2920	2951	2966	2997	3031	2915	3026	2936	2943
	1484	1487	1476	1486	1567	1482	1595	1494	1470
	1392	1393	1380	1367	1365	1380	1354	1392	1365
	1316	1323	1281	1278	1291	1321	1284	1336	1252
	1169	1184	1185	1177	1134	1175	1115	1177	1168
	976	978	972	950	937	978	929	984	952
	939	937	917	905	931	917	922	938	902
	830	831	819	823	784	828	815	824	819
	724	750	736	735	689	733	691	730	732
	517	513	532	515	530	499	530	510	521
	346	355	329	327	285	361	266	354	348
	329	333	284	288	228	327	235	321	285
	221	238	237	229	148	240	194	232	246
	131	106	167	176	127	119	134	114	179
	90	89	135	121	103	87	107	104	129
	52	47	93	107	62	79	98	68	101
$*OCHCH_3$	3047	3055	3061	3091	3087	3099	3065	3097	3051
	3019	3002	3020	3026	3006	3012	2985	3014	2980
	2932	2926	2936	2945	2936	2946	2910	2892	2909
	2894	2922	2912	2822	2861	2868	2785	2884	2827
	1655	1647	1635	1619	1638	1629	1711	1606	1610
	1395	1404	1405	1407	1403	1402	1406	1398	1407
	1385	1381	1382	1392	1394	1392	1392	1372	1382
	1370	1372	1364	1352	1364	1362	1363	1351	1368
	1316	1317	1312	1314	1316	1317	1316	1303	1301
	1109	1106	1103	1098	1106	1109	1098	1099	1067
	1070	1067	1066	1046	1058	1065	1072	1047	1056
	881	903	891	891	880	885	875	879	938
	729	712	729	714	736	750	736	692	696
	520	524	525	510	513	527	497	498	511
	234	243	176	214	211	210	191	232	175
	223	221	133	171	174	189	142	141	171
	116	131	104	124	141	133	87	95	134
	88	105	101	105	109	126	78	82	103
	77	83	71	86	98	92	67	40	91
	64	61	42	56	79	45	42	–	67
	–	–	–	38	29	–	–	–	10

Supplementary Table 8: PBE-D2 vibrational frequencies (in cm^{-1}) calculated for *OCCHO on different actives sites (Supplementary Figure 15). Vibrational modes can be visualized in the ioChem-BD database (see Supplementary Figure 17 for details).[16] For instance, vibrational modes of adsorbates on Cu(100) are accessible at Ref. [25].

Model	Oxide-derived copper								Cu(100)
$N_{Cu\text{-}Cu}$	4.46	5.00	6.17	6.49	6.97	7.48	8.03	9.42	8.00
*OCCHO	2946	2958	2946	2954	2964	2945	2955	3010	2965
	1502	1408	1409	1455	1428	1452	1473	1335	1326
	1458	1301	1297	1303	1301	1312	1307	1235	1266
	1296	1255	1204	1252	1238	1213	1272	1199	1149
	895	1007	999	996	1015	1035	980	1057	994
	795	790	811	833	819	843	811	764	831
	556	578	580	584	570	595	561	585	563
	436	498	484	484	478	499	453	457	475
	297	368	306	294	313	316	287	385	378
	251	310	259	283	277	297	274	295	284
	214	281	253	222	265	263	237	261	258
	207	264	220	210	225	230	214	229	215
	121	178	176	173	156	171	148	205	179
	100	133	147	135	134	137	133	185	139
	85	114	111	118	111	103	107	106	98

Supplementary Table 9: PBE-D2 vibrational frequencies (in cm^{-1}) calculated for *OHCCOH on different actives sites (Supplementary Figure 15). Vibrational modes can be visualized in the ioChem-BD database (see Supplementary Figure 17 for details).[16] For instance, vibrational modes of adsorbates on Cu(100) are accessible at Ref. [26].

Model	Oxide-derived copper								Cu(100)
$N_{Cu\text{-}Cu}$	4.46	5.00	6.17	6.49	6.97	7.48	8.03	9.42	8.00
*OHCCOH	3700	3596	3570	3686	3688	3744	3644	3741	3691
	3589	3023	3541	3543	3580	3475	3577	3484	3536
	1344	2040	1285	1322	1341	2037	1389	1287	1355
	1284	1350	1266	1293	1294	1568	1285	1261	1299
	1156	1226	1172	1159	1166	1235	1180	1090	1168
	1062	1170	1014	1056	1065	691	1071	967	1058
	986	818	835	973	977	557	975	885	986
	754	547	709	748	750	529	749	710	758
	563	470	609	581	556	484	542	611	589
	468	345	466	511	481	347	449	501	506
	337	333	370	370	355	316	347	335	371
	319	286	328	350	345	294	319	309	314
	302	253	288	292	300	247	287	271	298
	241	185	268	253	251	229	226	232	244
	224	174	247	210	225	184	188	221	216
	158	88	166	148	143	90	122	157	147
	100	56	150	97	104	72	96	134	99
	86	49	94	79	63	49	47	110	72

Supplementary Table 10: PBE-D2 vibrational frequencies (in cm^{-1}) calculated for *OCCHOH on different actives sites (Supplementary Figure 15). Vibrational modes can be visualized in the ioChem-BD database (see Supplementary Figure 17 for details).[16] For instance, vibrational modes of adsorbates on Cu(100) are accessible at Ref. [27].

Model	Oxide-derived copper								Cu(100)
$N_{Cu\text{-}Cu}$	4.46	5.00	6.17	6.49	6.97	7.48	8.03	9.42	8.00
*OCCHOH	3569	3610	3497	3563	3587	3461	3551	3576	3538
	3164	3076	3104	3161	3126	3133	3155	3006	3206
	1433	1398	1422	1441	1399	1434	1438	1354	1470
	1317	1319	1281	1293	1310	1288	1302	1299	1310
	1228	1228	1187	1227	1227	1222	1232	1205	1214
	1099	1108	1078	1104	1105	1123	1109	1090	1097
	1009	1002	944	1000	998	1026	1016	976	944
	778	775	775	783	780	792	778	778	783
	762	717	763	770	759	764	757	695	772
	585	512	636	617	539	663	595	515	643
	501	505	503	497	503	497	480	485	526
	355	375	356	382	311	369	327	345	348
	277	314	304	241	306	233	239	265	262
	229	224	223	222	234	220	216	205	236
	193	194	181	183	152	183	155	184	233
	148	124	178	137	135	153	131	132	193
	65	112	104	72	102	89	86	125	87
	61	77	75	43	73	62	65	95	31

Supplementary Table 11: PBE-D2 vibrational frequencies (in cm^{-1}) calculated for *CHO and *CH on different actives sites (Supplementary Figure 15). Vibrational modes can be visualized in the ioChem-BD database (see Supplementary Figure 17 for details).[16] For instance, vibrational modes of adsorbates on Cu(100) are accessible at Refs. [28,29].

Model	Oxide-derived copper								Cu(100)
N_{Cu-Cu}	4.46	5.00	6.17	6.49	6.97	7.48	8.03	9.42	8.00
*CHO	2766	2737	2781	2804	1729	2875	2699	2806	2876
	1686	1685	1489	1482	1648	1366	1566	1389	1227
	1199	1198	1254	1261	1167	1250	1181	1251	1130
	688	670	687	682	515	582	596	582	547
	454	456	467	470	348	455	381	418	441
	197	204	281	299	236	287	177	292	278
	131	134	198	188	184	229	158	229	232
	49	49	184	164	119	207	89	150	174
	42	11	75	78	75	133	54	109	164
*CH	2914	2973	2939	3029	3003	2926	2970	2995	2932
	651	725	631	620	695	635	683	754	680
	611	689	572	609	687	610	677	745	675
	506	455	445	532	510	523	486	449	472
	376	440	381	438	482	386	449	422	469
	302	424	336	365	363	358	379	386	394

Supplementary Table 12: PBE-D2 vibrational frequencies (in cm^{-1}) calculated for $*O^{13}CH^{13}CH_2$ and $*O^{13}CH^{13}CH_3$ on different actives sites (Supplementary Figure 15). Vibrational modes can be visualized in the ioChem-BD database (see Supplementary Figure 17 for details).[16] Vibrational modes on Cu(100) are accessible at Refs. [30,31].

Model	Oxide-derived copper								Cu(100)
N_{Cu-Cu}	4.46	5.00	6.17	6.49	6.97	7.48	8.03	9.42	8.00
$*OCHCH_2$	3114	3112	3123	3098	3165	3105	3155	3099	3139
	3010	3021	3022	3037	3070	3004	3047	3007	3034
	2913	2943	2956	2993	3022	2907	3019	2929	2935
	1447	1451	1443	1449	1519	1445	1543	1456	1438
	1380	1381	1367	1357	1356	1369	1345	1380	1353
	1312	1317	1270	1267	1284	1317	1278	1332	1240
	1143	1156	1158	1151	1112	1148	1092	1149	1143
	968	969	963	942	932	970	921	975	943
	929	926	908	896	922	907	917	927	895
	827	827	814	819	777	826	807	820	816
	720	745	731	729	683	727	684	725	727
	512	507	525	509	526	493	524	504	516
	338	351	322	324	282	358	264	344	342
	324	325	277	280	225	319	234	317	278
	219	234	232	223	146	234	189	230	242
	128	107	161	174	121	117	132	113	177
	93	88	127	117	100	87	107	106	127
	53	48	94	109	56	73	94	66	92
$*OCHCH_3$	3036	3043	3050	3080	3077	3088	3053	3087	3040
	3010	2991	3010	3016	2995	3003	2974	3005	2974
	2929	2919	2933	2939	2929	2943	2908	2884	2901
	2886	2913	2899	2812	2853	2858	2773	2877	2823
	1616	1607	1596	1577	1598	1590	1662	1564	1571
	1391	1399	1401	1404	1401	1399	1403	1394	1403
	1383	1380	1381	1390	1392	1389	1391	1369	1379
	1366	1367	1356	1346	1359	1356	1361	1347	1364
	1307	1308	1303	1305	1306	1306	1305	1293	1293
	1085	1080	1078	1076	1082	1084	1073	1077	1049
	1052	1048	1050	1029	1040	1047	1054	1028	1035
	869	889	877	875	865	871	857	864	921
	728	709	727	712	733	747	733	687	691
	514	515	517	504	505	522	490	491	505
	232	240	180	212	210	207	187	231	175
	226	219	131	169	169	185	136	133	171
	113	123	109	124	139	134	79	111	134
	94	112	96	101	110	125	72	83	103
	78	82	71	77	97	93	67	40	87
	52	48	44	42	75	57	35	–	66
	–	42	–	27	66	3	–	–	–

Supplementary Table 13: PBE-D2 vibrational frequencies (in cm^{-1}) calculated for $*H_2O$, *OH, and *O on different actives sites (Supplementary Figure 15). Vibrational modes can be visualized in the ioChem-BD database (see Supplementary Figure 17 for details).[16] Vibrational modes of adsorbates on Cu(100) are accessible at Refs. [32–34].

Model	Oxide-derived copper								Cu(100)
$N_{Cu\text{-}Cu}$	4.46	5.00	6.17	6.49	6.97	7.48	8.03	9.42	8.00
$*H_2O$	3766	3764	3767	3739	3797	3712	3694	3610	3643
	3594	3659	3479	3607	3689	3594	3569	3366	3529
	1527	1571	1517	1562	1563	1553	1549	1525	1556
	465	507	503	512	470	497	507	628	474
	286	419	294	435	388	445	479	534	438
	212	243	172	234	223	192	196	258	157
	192	103	117	159	85	85	116	183	136
	67	57	93	89	38	69	86	127	97
	5	12	–	65	–	31	60	85	–
*OH	3717	3725	3685	3730	3713	3723	3690	3712	3671
	706	718	732	667	712	640	625	786	620
	483	504	497	547	463	571	544	444	616
	372	387	364	405	335	348	377	364	300
	313	318	312	291	290	270	279	314	139
	94	108	116	110	155	225	93	116	126
*O	422	523	393	436	428	422	430	487	339
	396	449	301	401	422	403	409	361	338
	353	51	251	337	303	352	378	271	310

Supplementary Table 14: PBE-D2 vibrational frequencies (in cm^{-1}) calculated for *CO on different actives sites (Supplementary Figure 15). Vibrational modes can be visualized in the ioChem-BD database (see Supplementary Figure 17 for details).[16] For instance, vibrational modes of adsorbates on Cu(100) are accessible at Ref. [35].

Model	Oxide-derived copper								Cu(100)
$N_{Cu\text{-}Cu}$	4.46	5.00	6.17	6.49	6.97	7.48	8.03	9.42	8.00
*CO	2042	2047	2032	1893	1794	1890	1991	1768	1872
	345	342	342	311	306	322	326	299	289
	286	278	280	288	270	294	284	274	288
	283	277	272	253	247	257	252	232	236
	75	77	55	140	143	163	60	166	156
	60	58	54	56	122	53	41	128	57

Supplementary Table 15: Cu-Cu distances for atoms (1-10) within the coordination shell of the adsorbing site. AD stands for adatom. Structural models are reported in Supplementary Figure 15 and Supplementary Figure 23.

Site	N_{Cu-Cu}	1	2	3	4	5	6	7	8	9	10
Cu(111)-2AD	4.00	2.41	2.42	2.44	2.46	–	–	–	–	–	–
Cu(211)-2AD	4.00	2.39	2.42	2.45	2.47	–	–	–	–	–	–
Cu(100)-2AD	5.00	2.43	2.43	2.46	2.46	2.48	–	–	–	–	–
Cu(111)-3AD	5.00	2.44	2.44	2.44	2.48	2.48	–	–	–	–	–
Cu(711)-2AD	5.00	2.42	2.44	2.45	2.46	2.47	–	–	–	–	–
Cu(100)-4AD	6.00	2.45	2.47	2.47	2.48	2.50	2.50	–	–	–	–
Cu(310)	6.00	2.46	2.47	2.47	2.52	2.52	2.56	–	–	–	–
Cu(711)	7.00	2.47	2.47	2.51	2.51	2.53	2.56	2.56	–	–	–
Cu(110)	7.00	2.50	2.51	2.51	2.51	2.51	2.57	2.57	–	–	–
Cu(211)	7.00	2.48	2.48	2.51	2.51	2.51	2.56	2.56	–	–	–
Cu(533)	7.00	2.45	2.50	2.50	2.52	2.52	2.56	2.56	–	–	–
Cu(100)	8.00	2.53	2.53	2.53	2.53	2.57	2.57	2.57	2.57	–	–
Cu(111)	9.00	2.54	2.54	2.54	2.57	2.57	2.57	2.57	2.57	2.57	–
distorted site 1	4.46	2.34	2.45	2.51	–	–	–	–	–	–	–
distorted site 2	5.00	2.34	2.48	2.57	2.60	2.60	–	–	–	–	–
distorted site 3	6.17	2.41	2.47	2.47	2.53	2.59	2.63	–	–	–	–
distorted site 4	6.49	2.46	2.46	2.48	2.48	2.53	2.56	–	–	–	–
distorted site 5	6.97	2.37	2.43	2.46	2.48	2.49	2.50	2.63	–	–	–
distorted site 6	7.48	2.38	2.40	2.41	2.45	2.46	2.53	2.58	–	–	–
distorted site 7	8.03	2.46	2.50	2.56	2.56	2.58	2.59	2.62	2.62	–	–
distorted site 8	9.42	2.39	2.40	2.48	2.50	2.51	2.57	2.60	2.60	2.63	2.68

Supplementary Table 16: DFT adsorption energy (in eV) for *CO adsorption on atop sites on well-defined crystalline domains taken from Ref. [36] and distorted morphologies from Ref.[8]. Relative coordination numbers, localized 3D strains, d- and s-band center (vs Fermi energy) have been calculated according to Supplementary Eqs. (3)-(6). AD stands for adatom.

Site	$N_{Cu\text{-}Cu}$	3D Strain (%)	$\Sigma(\Delta d/d)\ N_{Cu\text{-}Cu}^{-1}$ (%)	$\varepsilon_d - \varepsilon_F$ / eV	$\varepsilon_s - \varepsilon_F$ / eV	$\Delta E_{*CO\text{-}atop}$
Cu(111)-2AD	4.00	21.30	5.32	–1.59	+0.79	–0.42
Cu(211)-2AD	4.00	21.30	5.32	–1.82	–0.24	–0.39
Cu(100)-2AD	5.00	22.83	4.57	–1.81	+0.23	–0.29
Cu(111)-3AD	5.00	22.05	4.41	–1.73	+0.49	–0.37
Cu(711)-2AD	5.00	23.60	4.72	–2.00	–0.97	–0.34
Cu(100)-4AD	6.00	21.24	3.54	–1.95	–0.25	–0.26
Cu(310)	6.00	16.18	2.70	–1.12	+3.28	–0.30
Cu(711)	7.00	14.60	2.09	–2.04	–0.47	–0.22
Cu(110)	7.00	11.93	1.70	–1.94	+0.35	–0.34
Cu(211)	7.00	14.60	2.09	–1.94	+0.51	–0.31
Cu(533)	7.00	14.60	2.09	–2.25	–2.89	–0.24
Cu(100)	8.00	6.12	0.76	–1.81	+1.17	–0.24
Cu(111)	9.00	3.42	0.38	–1.88	+1.26	–0.18
distorted site 1	4.46	15.87	3.56	–2.22	–3.33	–0.29
distorted site 2	5.00	12.37	2.47	–2.06	–3.03	–0.33
distorted site 3	6.17	15.46	2.50	–2.25	–3.53	–0.13
distorted site 4	6.49	17.35	2.67	–2.32	–3.54	–0.18
distorted site 5	6.97	26.69	3.83	–2.51	–4.01	–0.01
distorted site 6	7.48	30.58	4.09	–2.61	–4.23	+0.03
distorted site 7	8.03	7.67	0.96	–2.39	–3.75	+0.01
distorted site 8	9.42	22.02	2.34	–2.62	–4.43	+0.08

Supplementary Table 17: Fit parameters of correlations reported in Supplementary Figure 25.

Correlation of $\varepsilon_s - \varepsilon_F$ vs $\varepsilon_d - \varepsilon_F$	a (eV)	b (–)	R^2	χ^2
Distorted domains	+1.8 ± 0.3	+2.34 ± 0.14	0.97	0.03
Crystalline domains	+9 ± 1	+4.6 ± 0.8	0.74	5.6

Supplementary Table 18. C-C bond length for different C_{2+} intermediates. Typical values are 1.54 Å for a single bond, 1.34 Å for a double bond, 1.20 Å for a triple bond.

$N_{Cu\text{-}Cu}$	$\Sigma(\Delta d/d)\ N_{Cu\text{-}Cu}^{-1}$ (%)	C-C bond length / pm						
		*COCO	*COCOH	*OCCHO	*HOCCOH	*OCCHOH	*OCHCH$_2$	*OCHCH$_3$
4.46	3.56	–	1.50	1.50	1.41	1.40	1.41	1.48
5.00	2.47	1.34	1.29	1.46	1.26	1.41	1.41	1.48
6.17	2.50	–	1.34	1.45	1.42	1.41	1.40	1.49
6.49	2.67	1.47	1.44	1.46	1.41	1.39	1.39	1.49
6.97	3.83	1.67	1.49	1.46	1.42	1.42	1.35	1.49
7.48	4.09	1.53	1.42	1.46	1.31	1.40	1.41	1.49
8.03	0.96	–	1.45	1.47	1.39	1.39	1.34	1.49
9.42	2.34	1.44	1.30	1.42	1.43	1.45	1.41	1.49
8.00	0.76	1.34	1.34	1.43	1.41	1.38	1.39	1.49

Supplementary Table 19: Fit parameters of correlations reported in Supplementary Figure 26.

Correlation of F.E.	a (%)	b (–)	R^2	χ^2
EtOH vs C_2H_4	–2.4 ± 1.5	+0.48 ± 0.08	0.90	2.53
Acetaldehyde vs EtOH	+0.04 ± 0.04	+0.027 ± 0.006	0.82	0.004
1-PrOH vs Allyl Alcohol	+0.5 ± 0.3	+1.9 ± 0.3	0.94	0.20

Supplementary References

1. Schouten, K. J. P., van der Ham, C. J. M., Qin, Z. & Koper, M. T. M. A new mechanism for the selectivity to C_1 and C_2 species in the electrochemical reduction of carbon dioxide on copper electrodes. Chem. Sci. 2, 1902 (2011).
2. Delmo, E. P. et al. The Role of Glyoxal as an Intermediate in the Electrochemical CO_2 Reduction Reaction on Copper. The Journal of Physical Chemistry C 127, 4496–4510 (2023).
3. Reichert, A. M., Piqué, O., Parada, W. A., Katsounaros, I. & Calle-Vallejo, F. Mechanistic insight into electrocatalytic glyoxal reduction on copper and its relation to CO_2 reduction. Chem Sci 13, 11205–11214 (2022).
4. Kuhl, K. P., Cave, E. R., Abram, D. N. & Jaramillo, T. F. New insights into the electrochemical reduction of carbon dioxide on metallic copper surfaces. Energy Environ. Sci. 5, 7050–7059 (2012).
5. Garza, A. J., Bell, A. T. & Head-Gordon, M. Mechanism of CO_2 reduction at copper surfaces: Pathways to C_2 products. ACS Catal. 8, 1490–1499 (2018).
6. Birdja, Y. Y. et al. Advances and challenges in understanding the electrocatalytic conversion of carbon dioxide to fuels. Nat Energy 4, 732–745 (2019).
7. Mavrikakis, M., Hammer, B. & Nørskov, J. K. Effect of strain on the reactivity of metal surfaces. Phys Rev Lett 81, 2819–2822 (1998).
8. Bernal, M. et al. CO_2 electroreduction on copper-cobalt nanoparticles: Size and composition effect. Nano Energy 53, 27–36 (2018).
9. Zhan, C. et al. Revealing the CO coverage-driven C–C coupling mechanism for electrochemical CO_2 reduction on Cu_2O nanocubes via Operando Raman spectroscopy. ACS Catal. 11, 7694–7701 (2021).
10. Dattila, F., García-Muelas, R. & López, N. Active and Selective Ensembles in Oxide-Derived Copper Catalysts for CO_2 Reduction. ACS Energy Lett. 5, 3176–3184 (2020).
11. Álvarez-Moreno, M. et al. Managing the computational chemistry big data problem: The ioChem-BD platform. J Chem Inf Model 55, 95–103 (2015).
12. Yang, Y. et al. Operando studies reveal active Cu nanograins for CO_2 electroreduction. Nature 614, 262–269 (2023).
13. Sebastián-Pascual, P. & Escudero-Escribano, M. Surface characterization of copper electrocatalysts by lead underpotential deposition. J. Electroanal. Chem. 896, 115446 (2021).
14. Li, J. et al. Intercepting Elusive Intermediates in Cu-Mediated CO Electrochemical Reduction with Alkyl Species. J Am Chem Soc 144, 20495–20506 (2022).
15. Chang, X., Malkani, A., Yang, X. & Xu, B. Mechanistic Insights into Electroreductive C–C Coupling between CO and Acetaldehyde into Multicarbon Products. J Am Chem Soc 142, 2975–2983 (2020).
16. Dattila, F. Raman-C2. ioChem-BD https://doi.org/10.19061/iochem-bd-1-251 (2024) doi:10.19061/iochem-bd-1-251.
17. Dattila, F. Cu(100)-p(3×3)-CO_3. ioChem-BD https://iochem-bd.iciq.es/browse/cml2html/100/40740/output.cml (2024).
18. Dattila, F. Cu(100)-p(3×3)-HCO_2. ioChem-BD https://iochem-bd.iciq.es/browse/cml2html/100/40742/output.cml (2024).
19. Dattila, F. Cu(100)-p(3×3)-HCOOH. ioChem-BD https://iochem-bd.iciq.es/browse/cml2html/100/40741/output.cml (2024).
20. Dattila, F. Cu(100)-p(3×3)-COCO. ioChem-BD https://iochem-bd.iciq.es/browse/cml2html/100/40737/output.cml (2024).

21. Dattila, F. Cu(100)-p(3×3)-COCOH. ioChem-BD https://iochem-bd.iciq.es/browse/cml2html/100/40736/output.cml (2024).
22. Dattila, F. Cu(100)-p(3×3)-$COCO_2$. ioChem-BD https://iochem-bd.iciq.es/browse/cml2html/100/40735/output.cml (2024).
23. Dattila, F. Cu(100)-p(3×3)-$OCHCH_2$. ioChem-BD https://iochem-bd.iciq.es/browse/cml2html/100/40738/output.cml (2024).
24. Dattila, F. Cu(100)-p(3×3)-$OCHCH_3$. ioChem-BD https://iochem-bd.iciq.es/browse/cml2html/100/40732/output.cml (2024).
25. Dattila, F. Cu(100)-p(3×3)-OCCHO. ioChem-BD https://iochem-bd.iciq.es/browse/cml2html/100/40729/output.cml (2024).
26. Dattila, F. Cu(100)-p(3×3)-HOCCOH. ioChem-BD https://iochem-bd.iciq.es/browse/cml2html/100/40731/output.cml (2024).
27. Dattila, F. Cu(100)-p(3×3)-OCCHOH. ioChem-BD https://iochem-bd.iciq.es/browse/cml2html/100/40728/output.cml (2024).
28. Dattila, F. Cu(100)-p(3×3)-CHO. ioChem-BD https://iochem-bd.iciq.es/browse/cml2html/100/40730/output.cml (2024).
29. Dattila, F. Cu(100)-p(3×3)-CH. ioChem-BD https://iochem-bd.iciq.es/browse/cml2html/100/40744/output.cml (2024).
30. Dattila, F. Cu(100)-p(3×3)-$OCHCH_2$-^{13}C. ioChem-BD https://iochem-bd.iciq.es/browse/cml2html/100/68201/output.cml (2024).
31. Dattila, F. Cu(100)-p(3×3)-OCHCH3-^{13}C. ioChem-BD https://iochem-bd.iciq.es/browse/cml2html/100/68199/output.cml (2024).
32. Dattila, F. Cu(100)-p(3×3)-H_2O. ioChem-BD https://iochem-bd.iciq.es/browse/cml2html/100/40745/output.cml (2024).
33. Dattila, F. Cu(100)-p(3×3)-OH. ioChem-BD https://iochem-bd.iciq.es/browse/cml2html/100/40746/output.cml (2024).
34. Dattila, F. Cu(100)-p(3×3)-O. ioChem-BD https://iochem-bd.iciq.es/browse/cml2html/100/40747/output.cml (2024).
35. Dattila, F. Cu(100)-p(3×3)-CO. ioChem-BD https://iochem-bd.iciq.es/browse/cml2html/100/40743/output.cml (2024).
36. Calle-Vallejo, F., Loffreda, D., Koper, M. T. M. & Sautet, P. Introducing structural sensitivity into adsorption-energy scaling relations by means of coordination numbers. Nat Chem 7, 403–410 (2015).
